# Genome-Wide Association Study of Resistance to Soybean Cyst Nematode (*Heterodera glycines*) HG Type 2.5.7 in Wild Soybean (*Glycine soja*)

**DOI:** 10.3389/fpls.2016.01214

**Published:** 2016-08-17

**Authors:** Hengyou Zhang, Chunying Li, Eric L. Davis, Jinshe Wang, Joshua D. Griffin, Janice Kofsky, Bao-Hua Song

**Affiliations:** ^1^Bao-Hua Song Lab, Department of Biological Sciences, University of North Carolina at CharlotteCharlotte, NC, USA; ^2^Eric Davis Lab, Department of Plant Pathology, North Carolina State UniversityRaleigh, NC, USA; ^3^Institute of Industrial Crops, Henan Academy of Agricultural Sciences, National Subcenter for Soybean Improvement/Key Laboratory of Oil CropsZhengzhou, China; ^4^SAS InstituteCary, NC, USA

**Keywords:** genome-wide association analysis, soybean cyst nematode, SCN, wild soybean, *Glycine soja*, resistance

## Abstract

Soybean cyst nematode (SCN) is the most destructive soybean pest worldwide. Host plant resistance is the most environmentally friendly and cost-effective way of mitigating SCN damage to soybeans. However, overuse of the resistant soybean [*Glycine max* (L.) Merr.] cultivars from limited genetic resources has resulted in SCN race shifts in many soybean-growing areas. Thus, exploration of novel sources of SCN resistance and dissection of the genetic basis are urgently needed. In this study, we screened 235 wild soybean (*Glycine soja* Sieb. & Zucc.) accessions to identify genotypes resistant to SCN HG Type 2.5.7 (race 5), a less investigated type but is prevalent in the southeastern US. We also dissected the genetic basis of SCN resistance using a genome-wide association study with SNPs genotyped by SoySNP50k iSelect BeadChip. In total, 43 resistant accessions (female index < 30) were identified, with 10 SNPs being significantly associated with SCN HG 2.5.7 resistance in this wild species. Furthermore, four significant SNPs were localized to linked regions of the known quantitative trait locus (QTL) *rhg1* on chromosome 18. The other four SNPs on chromosome 18 and two SNPs on chromosome 19 are novel. Genes encoding disease resistance-related proteins with a leucine-rich region, a mitogen-activated protein kinase (MAPK) on chromosome 18, and a MYB transcription factor on chromosome 19 were identified as promising candidate genes. The identified SNPs and candidate genes will benefit future marker-assisted breeding and dissection of the molecular mechanisms underlying the soybean-SCN interaction.

## Introduction

Soybean [*Glycine max* (L.) Merr.] is one of the most important economic crops. It has been cultivated for thousands of years and provides protein for humans and animal consumption. However, soybean plants are challenged by soybean cyst nematode (SCN, *Heterodera glycines* Ichinohe). SCN is the most devastating pest that causes severe soybean [*Glycine max* (L.) Merr.] yield loss worldwide. It also has been demonstrated that SCN suppresses seed yield more than any other single soybean pathogen (Wrather and Koenning, 2006). Although nematicides have shown some short-term efficacy for SCN management, they are costly and not environmentally friendly. Rotations to non-host crops are effective but dependent upon the profitability and practicality of the non-host crop. In contrast, the development and use of resistant soybean cultivars is a cost-effective and environmentally-friendly means of managing SCN. Continuous use of a single resistant cultivar in a certain field generally drives the SCN population to evolve to overcome host resistance, however, diminishing the utility of any given SCN-resistant soybean cultivar.

The variability in SCN populations was developed into a scheme of 16 potential races of SCN based on four soybean genotypes (which actually represent three genetic sources of SCN resistance; Riggs and Schmitt, 1988). Because the term “race” is inappropriate for characterization of a heterogeneous population of SCN genotypes, a system for identifying SCNs by HG Type was developed using seven independent sources of soybean resistance to SCN (Niblack et al., 2002). Although HG Type is a superior measure of SCN variability, the soybean-breeding efforts over past decades have often documented SCN race; that terminology is occasionally used here only for direct comparison to previous breeding and mapping reports. The majority of available soybean cultivars have sources of resistance to SCN races 1 (HG Type 2) or 3 (HG Type 0), though, increased nematode reproduction by resistance-breaking SCN populations (Delheimer et al., 2010) has been observed for the even more recent soybean genotypes derived from PI437654 with resistance to “all races” of SCN (Anand, 1991). As all current sources of SCN resistance are derived from accessions of cultivated soybean (*G. max*), it seems appropriate to explore new sources of resistance to SCN in related wild species of soybean.

A key step in developing SCN-resistant varieties is elucidation of the genetic basis of resistance. To date, knowledge of the genetic basis of soybean SCN resistance is largely based on classical quantitative trait locus (QTL) mapping studies. Additionally, most research has focused on dissecting the mechanism of soybean resistance to races 1 and 3, which are prevalent in the central US. The identification of two QTLs, *rhg1* on chromosome 18, conferring HG Type 0 resistance (Cook et al., 2012), and *Rhg4* on chromosome 8, conferring HG Type 7 resistance (Liu et al., 2012), has suggested a race-specific resistance mechanism for the soybean-SCN interaction. However, race specificity is relatively unusual because cloning of the *rhg1* (Cook et al., 2012) locus indicated that rather canonical NB-LRR resistance genes (DeYoung and Innes, 2006), the copy number of three tandem genes (an amino acid transporter, an alpha-SNAP, and the WI12 protein) and *rhg-4* (a serine hydroxymethyltransferase; Liu et al., 2012) confer resistance to SCN.

SCN HG Type 2.5.7 is prevalent in the south-eastern US. Our understanding of soybean resistance to HG 2.5.7 is primarily based on linkage mapping using segregated populations. An early study by Anand and Raoarelli (1989) indicated that at least one gene controls soybean resistance to HG 2.5.7. Thus far, at least 22 QTLs distributed on 10 chromosomes (1, 5, 6, 8, 10, 11, 15, 18, 19, and 20) have been associated with resistance to HG 2.5.7 (race 5; Yue et al., 2001a,b; Guo et al., 2006a,b; Wu et al., 2009; Vuong et al., 2010; Abdelmajid et al., 2014). Among these QTLs, genomic regions either containing or adjacent to the *rhg1* locus on chromosome 18 have repeatedly been identified using different resistant soybean sources (Yue et al., 2001a; Guo et al., 2006a,b; Wu et al., 2009), suggesting the presence of highly important genes in this region that confer HG 2.5.7 resistance. In addition, the results of a previous study (Concibido et al., 2004) suggest that this region also confers broad-spectrum resistance to many other SCN HG types. Despite progress in identification of the QTLs underlying resistance to HG 2.5.7, pinpointing causal genes in large QTL regions and dissecting the molecular mechanisms underlying resistance to this HG type remain challenging.

The genome-wide association study (GWAS) strategy is considered an efficient and complementary approach to classical bi-parental QTL mapping for elucidating the genetic basis of complex trait variation (Sonah et al., 2015). By applying GWAS approach to cultivated soybean populations, the previously identified QTLs associated with SCN resistance have recently been verified, and novel candidate genes have been discovered in relatively less time (Han et al., 2015; Vuong et al., 2015). To date, large-scale screening of resistance responses to HG Type 0 and HG Type 1.2.3.5.7, followed by GWAS, has been conducted in cultivated soybean populations (Han et al., 2015; Vuong et al., 2015). Conversely, dissecting the genetic basis of resistance to SCN HG 2.5.7 applying GWAS strategy has not been reported for either cultivated populations or wild relatives.

*Glycine soja* Sieb. & Zucc. is the wild progenitor of cultivated soybean. During the domestication process, more than half of the genome-wide genetic diversity was lost (Zhou et al., 2015) in cultivated soybea. This genetic “bottleneck” in soybean has hindered the development of diverse soybean cultivars resistance to multiple races of SCN. To enrich the resistant gene pool, it is necessary to identify and utilize novel and exotic resources beyond the currently employed soybean cultivars. Compared with cultivated soybeans growing in a farmed field, *G. soja* experiences various environmental abiotic and biotic stresses. And thus, it holds great potential for developing varieties tolerant/resistant to environmental stress. In addition, *G. soja* and *G. max* are cross-compatible, producing vigorous and fertile F_1_ seeds; therefore, transfer of useful genes to soybean cultivars is efficient without use of biotechnology. A number of studies have shown the potential of employing *G. soja* to identify genes underlying tolerance to various abiotic (Qi et al., 2014) and biotic stresses (Winter et al., 2007; Kim et al., 2011). Regardless, only a few studies have applied *G. soja* to identify QTLs for resistance to HG 2.5.7 using traditional QTL mapping methods (Kim et al., 2011; Kim and Diers, 2013), whereas no studies have applied high-density single-nucleotide polymorphism (SNP) markers for understanding the genetic basis of SCN resistance to HG 2.5.7 with GWAS in *G. soja*.

In this study, we screened a set of 235 *G. soja* accessions with the aims of (1) identifying HG 2.5.7-resistant accessions in the *G. soja* population, (2) identifying genomic regions significantly associated with HG 2.5.7 resistance using genome-wide association mapping with SNPs genotyped by SoySNP50k iSelect BeadChip, and (3) identifying candidate genes involved in HG 2.5.7 resistance.

## Materials and methods

### Plant materials and SCN bioassay

**Figure 1:**
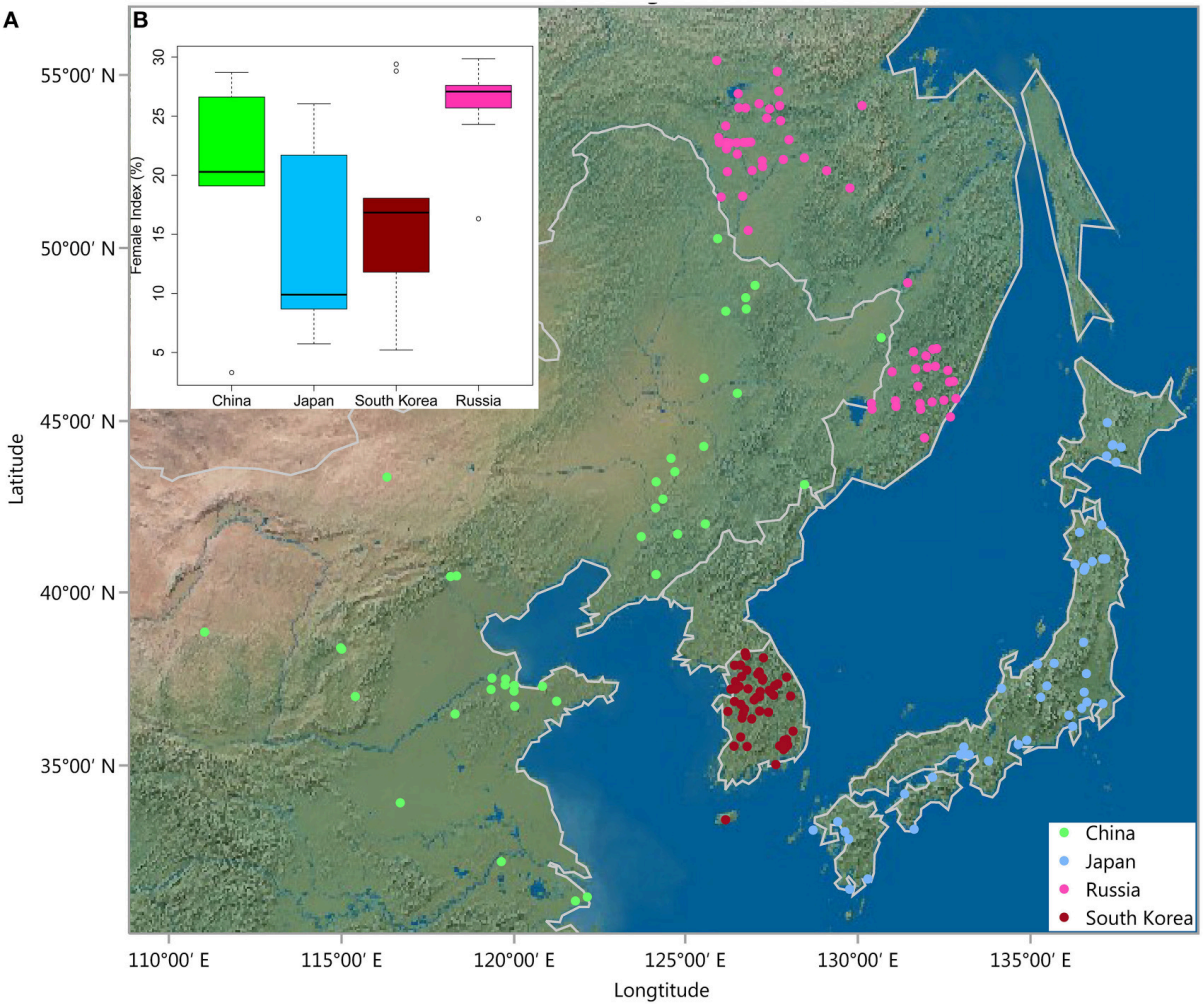
**(A)** Geographic distribution of 235 *G. soja* accessions in East Asia. **(B)** Box plot showing differences in female index (FI < 30) between four countries in East Asia.

A total of 235 *G. soja* accessions from USDA Soybean Germplasm Collection were used for resistance screening and association analysis (Table S1). This set of *G. soja* accessions was originally from four East Asian countries: China, Japan, Korea, and Russia (Figure 1A). SCN HG Type 2.5.7 was verified using HG type test indicator lines (Peking, PI88788, PI90763, PI437654, PI209332, PI89772, and PI548316) used in Niblack et al. (2002) before the large screening experiment. The verification results are shown in Table S2. SCN HG Type 2.5.7 has been maintained on susceptible soybean cv. Williams 82 in a greenhouse at the University of North Carolina, Charlotte for more than 30 generations.

Seeds of all accessions were first stratified and then germinated on germination paper for 3–4 days. Each seedling was then transplanted into a cone container (Stuewe & Sons, Tangent, Oregon, USA) containing a pasteurized soil/sand mixture. Four plants of each *G. soja* accession, seven soybean indicator lines and cv. Williams 82 as a susceptible control were used for inoculation. The plants were grown in a randomized complete block design in a greenhouse maintained at 27°C with 15 h of light per day. Two days after transplanting, the roots of each individual plant were inoculated with 2500 SCN eggs; the plants were watered regularly to maintain soil moisture. Female cysts were collected from the root samples and soil and counted under a stereomicroscope 35 days after inoculation. The average number of females of four replicates was used for calculation of the female index (FI) using the following equation: FI = (number of females on a given individual/average number of females on the susceptible control)/100 (Niblack et al., 2009).

### Genotype data and quality control

SNP data for the 235 *G. soja* accessions were retrieved from the publically available SoyBase website (http://soybase.org/snps/). Illumina SoySNP50k iSelect BeadChip (Illumina, San Diego, CA. USA) containing a total of 52,041 SNPs was used to genotype the USDA soybean and wild soybean germplasm, as described in previous studies (Song et al., 2013, 2015). SNPs without a physical position on any of the 20 *G. max* chromosomes or those with a minor allele frequency (MAF) < 0.05 were excluded from further analyses. Markers with a missing rate of > 10% were eliminated, and remaining missing data were imputed using BEAGLE (v 3.3.1; Browning and Browning, 2007, 2009).

### Population structure and linkage disequilibrium analysis

The population structure of the *G. soja* accessions was determined using principle component analysis (PCA) in the GAPIT software package (Lipka et al., 2012). A neighbor-joining (NJ) tree was constructed using TASSEL (Bradbury et al., 2007) and visualized with MEGA version 7.0 (Kumar et al., 2016). Pairwise linkage disequilibrium (LD) between SNP markers was calculated using squared allele frequency correlations (*r*^2^) with the R package synbreed (Wimmer et al., 2012). Only *r*^2^-values for SNPs with a pairwise distance less than 1 Mb from each chromosome were adopted to assess the average LD decay, as previously described (Zhou et al., 2015). The LD decay rate of the population was defined as the chromosome distance at which the average *r*^2^ decreased to half of its maximum value (Lam et al., 2010).

### Genome-wide association analysis

The association analysis was performed with Compressed Mixed Linear Model (cMLM) in GAPIT (Lipka et al., 2012) using the first three PCs and Kinship matrix to control the population structure. The threshold of significance for association was determined using the empirical significance values (*P* < 0.001) generated from 1000 permutations of association analyses, as previously described (Zhang et al., 2015b).

### Candidate gene prediction

Genes located within 50 kb on either side of significant SNPs were selected as possible candidate genes. The protein sequences encoded by the predicated genes were retrieved from the Phytozome database (https://phytozome.jgi.doe.gov). Functional annotation of the genes was conducted using BLAST2GO software with a BLASTp search (Conesa et al., 2005), as well as reference annotation of the soybean reference genome Wm82.a2.v1 (SoyBase, http://www.soybase.org) and previously published literature.

## Results

### Variation in resistance levels among *G. soja* accessions

Phenotypic evaluation revealed a broad range of SCN HG 2.5.7 resistance in the 235 *G. soja* accessions, with FI values from 3.2 to 277.4% (Figure S1). A total of 43 resistant accessions were identified, with eight showing a high level of resistance (0 < FI < 10) and 35 having moderate resistance to HG 2.5.7 (10 < FI < 30).

To investigate whether the resistance levels of *G. soja* are related to the original ecological distribution (Figure 1A), the FI values of the resistant accessions were plotted against their country of origin. As shown in Figure 1B, although great variation in FIs was observed, accessions from Japan and South Korea showed higher resistance levels on average compared with those from China and Russia. Upon close investigation of the data, we found that six of the eight highly resistant accessions (0 < FI < 10) were originally from Japan and the remaining two from China and South Korea.

### SNP data and LD analysis

A total of 41,087 SNPs were polymorphic in our data set; after quality control, 32,187 SNPs with an MAF ≥ 0.05 were used for further analyses. The SNP numbers ranged from 1235 on chromosome 20–2392 on chromosome 18, with an average of 1609 for each soybean chromosome. Accordingly, the SNP density across chromosomes ranged from 43.9 kb/SNP on chromosome 1–22.1 kb/SNP on chromosome 8, with an average genome-wide SNP density of one SNP per 30 kb (Figure S2).

**Figure 2:**
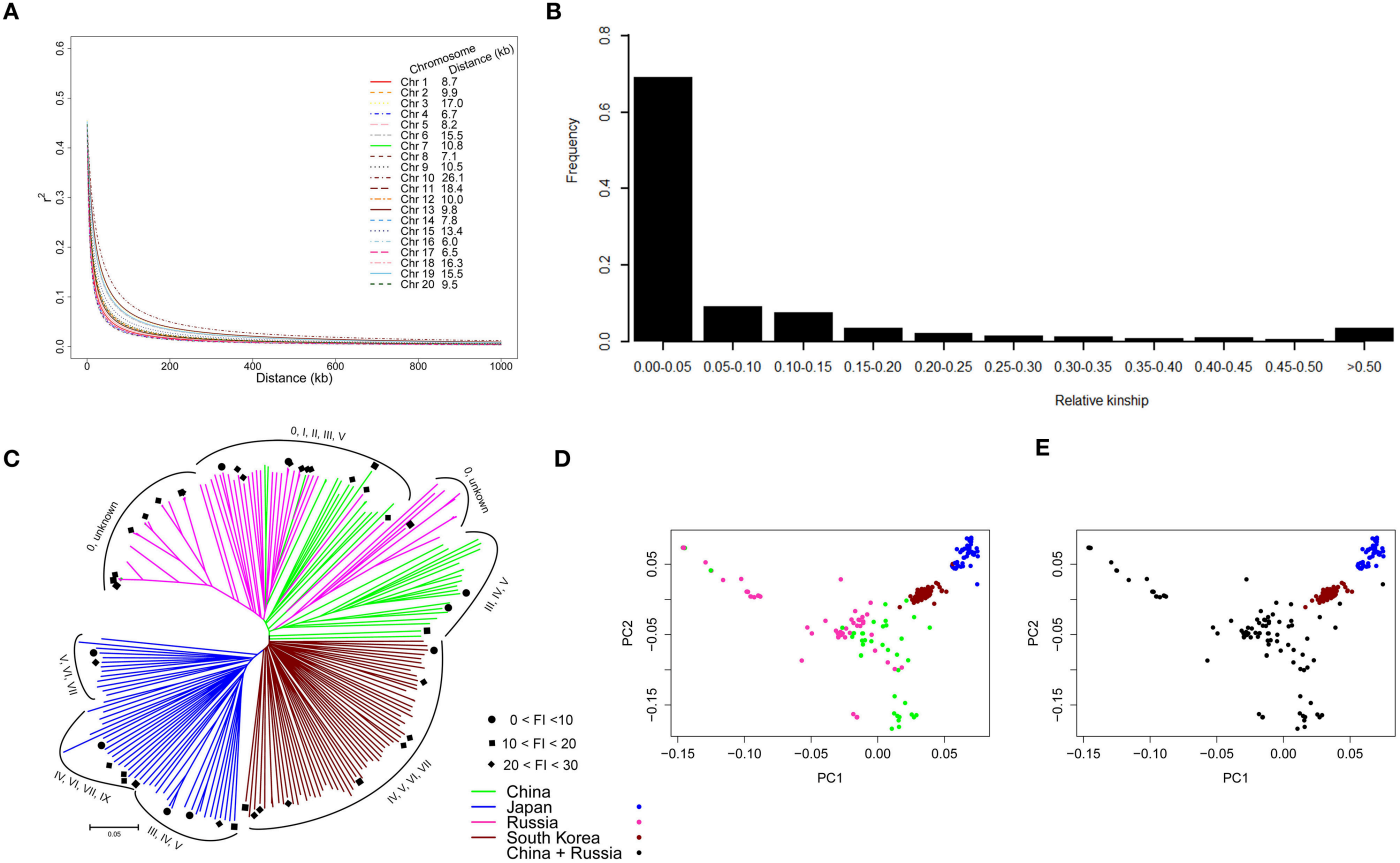
**Analysis of LD decay, relative kinship, and estimated population structure. (A)** LD decay pattern of SNPs on 20 soybean chromosomes. The distance (kb) of LD decay per chromosome is given beside the legend. **(B)** Distribution of pairwise kinship for 235 *G. soja* accessions. **(C)** Unrooted NJ tree of 235 *G. soja* accessions. The maturity group (MG) for each accession is labeled with Roman numerals based on the clusters. Resistant and moderately resistant accessions are marked with a solid circle (FI < 10), square (10 < FI < 20), and diamond (20 < FI < 30). **(D)** Plot of the first two PCs of the panel based on country of origin. **(E)** Plot of grouping results of the panel based on the first three PCs. The color-coded subpopulations indicated in **(C–E)** represent the country of origin.

To evaluate the mapping resolution for GWAS, the distribution of the average extent of LD decay as well as the *r*^2^-value between different physical distances on each chromosome were quantified. As shown in Figure 2A, LD decay was different on each chromosome, ranging from 6.0 kb on chromosome 16–26.1 kb on chromosome 10 (Figure 2A; Figure S3). The average LD decay for all chromosomes was estimated at 10 kb. Chromosome 18 ranked among the top five chromosomes showing the high levels of LD.

### Relative kinship and population structure

The relative kinship between any two *G. soja* accessions was estimated. As shown in Figure 2B, 69.1% of the pairwise kinship estimates between accessions ranged from 0 to 0.05, with a continuously decreasing number of pairs as kinship estimates increased in the estimate categories. This result revealed that most accessions in the panel had a null or weak kinship.

To further understand population stratification in our population, an NJ tree for the 235 *G. soja* accessions was constructed using 32,187 SNPs. As shown in Figure 2C, the resulting NJ tree classified the population into three large clusters, with accessions from Japan and South Korea each forming a cluster and accessions from China and Russia sharing one larger cluster. The high correlation between the geographical origin and population structure of the 235 accessions suggested that geographical origin is the main factor affecting the population structure of the panel. Based on the distribution of maturity groups (MGs) for each accession in the NJ tree, no obvious correlation was observed between population structure and MGs. We also conducted PCA for this association panel using the same SNP set. Figure 2D illustrates the consistent result, as shown in the NJ tree (Figure 2C), observed by referencing the country of origin. Furthermore, we found that the first three PCs, which explained 13.8% of the genetic variation, could effectively separate the association panel into three clusters (Figure 2E); this result is consistent with the NJ tree. Thus, we used the first three PCs to account for the observed population structure.

### Genome-wide association analyses

**Figure 3:**
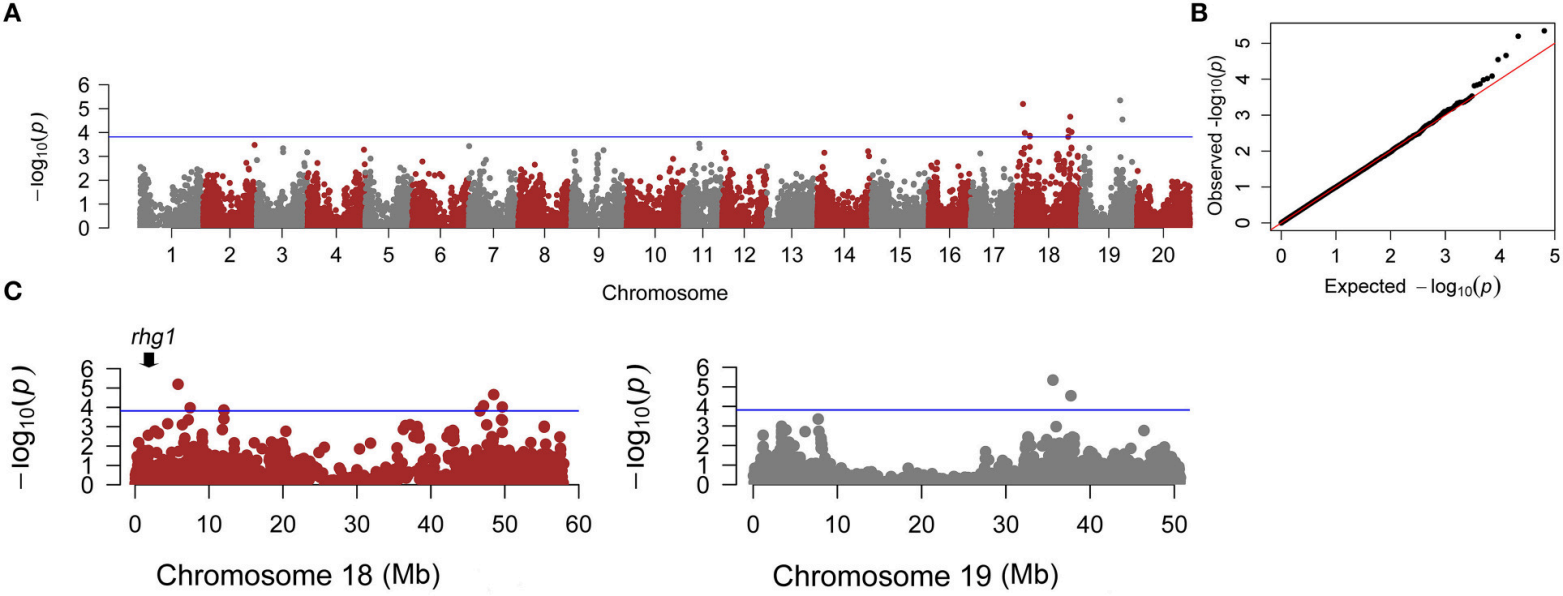
**Genome-wide association analyses (GWAS) for SCN resistance. (A)** Manhattan plot for SCN resistance. **(B)** Quantile-Quantile plot using cMLM. **(C)** Zoomed-in Manhattan plots for chromosomes 18 and 19. The blue line represents the threshold (*P* < 10^−3.81^) defined by 1000 permutations of the association analysis.

A total of 10 SNPs significantly associated with SCN resistance to HG Type 2.5.7 were identified by GWAS under cMLM, which controlled for population structure and familial relatedness (Table 1; Figure 3). Interestingly, the 10 significant SNPs were distributed on two chromosomes: 18 and 19. Of the 10 significant SNPs, eight could be grouped into two loci on chromosome 18. One locus containing four SNPs is located in the linked regions (5.8–12.0 Mb) of known *rhg1*, which were reported as conferring resistance to multiple SCN HG types (races), including HG Type 2.5.7 (previously named race 5; Yue et al., 2001a). The other locus on chromosome 18 is adjacent to the previously reported race 5 resistance QTL (SCN 20-4; Yue et al., 2001a). The other two SNPs are located on chromosome 19, from 35.5 to 37.8 Mb, a region that partially overlaps with a previously reported region conferring SCN resistance to HG Type 0 (race 3; Vuong et al., 2015). The 10 association SNPs explained 10.93–13.93% of the total phenotypic variation.

**Table 1:** **Significant SNPs and predicted candidate genes associated with SCN HG Type 2.5.7 resistance in ***G. soja*****.

**SNP**	**Chr**	**Position**	***P*-value**	**MAF**	***R*^2^ (%)**	**QTLs**	**Gene model**	**Functional description**
ss715631923	18	5,814,672	6.38E-06	0.15	13.62	Yue et al., 2001a	Glyma.18G063400	Pleiotropic drug resistance
							Glyma.18G063500	RING/U-box superfamily protein
							Glyma.18G064100	Calmodulin-binding receptor-like kinase
ss715632647	18	7,450,433	1.05E-04	0.06	11.24	Yue et al., 2001a	Glyma.18G077900	CC-NBS-LRR class disease resistance protein
							Glyma.18G078000	NB-ARC-containing disease resistance protein
ss715628640	18	12,004,584	1.36E-04	0.06	11.02	Yue et al., 2001a	Glyma.18G106800	Mitogen-activated protein kinase
ss715628650	18	12,044,370	1.45E-04	0.06	10.97	Yue et al., 2001a	Glyma.18G107000	Ribose-phosphate pyrophosphokinase
							Glyma.18G107100	Glycerol-3-phosphate acyltransferase
ss715631131	18	46,643,373	1.52E-04	0.06	10.93	Yue et al., 2001a	Glyma.18G193400	Laccase/Diphenol oxidase family protein
							Glyma.18G193800	LRR-RLP
ss715631193	18	47,125,551	8.21E-05	0.06	11.44	Yue et al., 2001a	Glyma.18G196200	mRNA capping enzyme family protein
ss715631383	18	48,495,551	2.19E-05	0.06	12.56	Yue et al., 2001a	Glyma.18G203500	Stress-induced protein
ss715631522	18	49,659,228	9.59E-05	0.14	11.31	Yue et al., 2001a	Glyma.18G210300	HEAT repeat; WD domain, G-beta repeat protein
							Glyma.18G210500	Pyrimidine 4
ss715634285	19	35,586,767	4.51E-06	0.08	13.93	–	–	–
ss715634622	19	37,724,122	2.85E-05	0.21	12.34	–	Glyma.19G120200	MATE efflux family protein
							Glyma.19G119300	MYB transcription factor

### Prediction of candidate genes for SCN resistance

**Figure 4:**
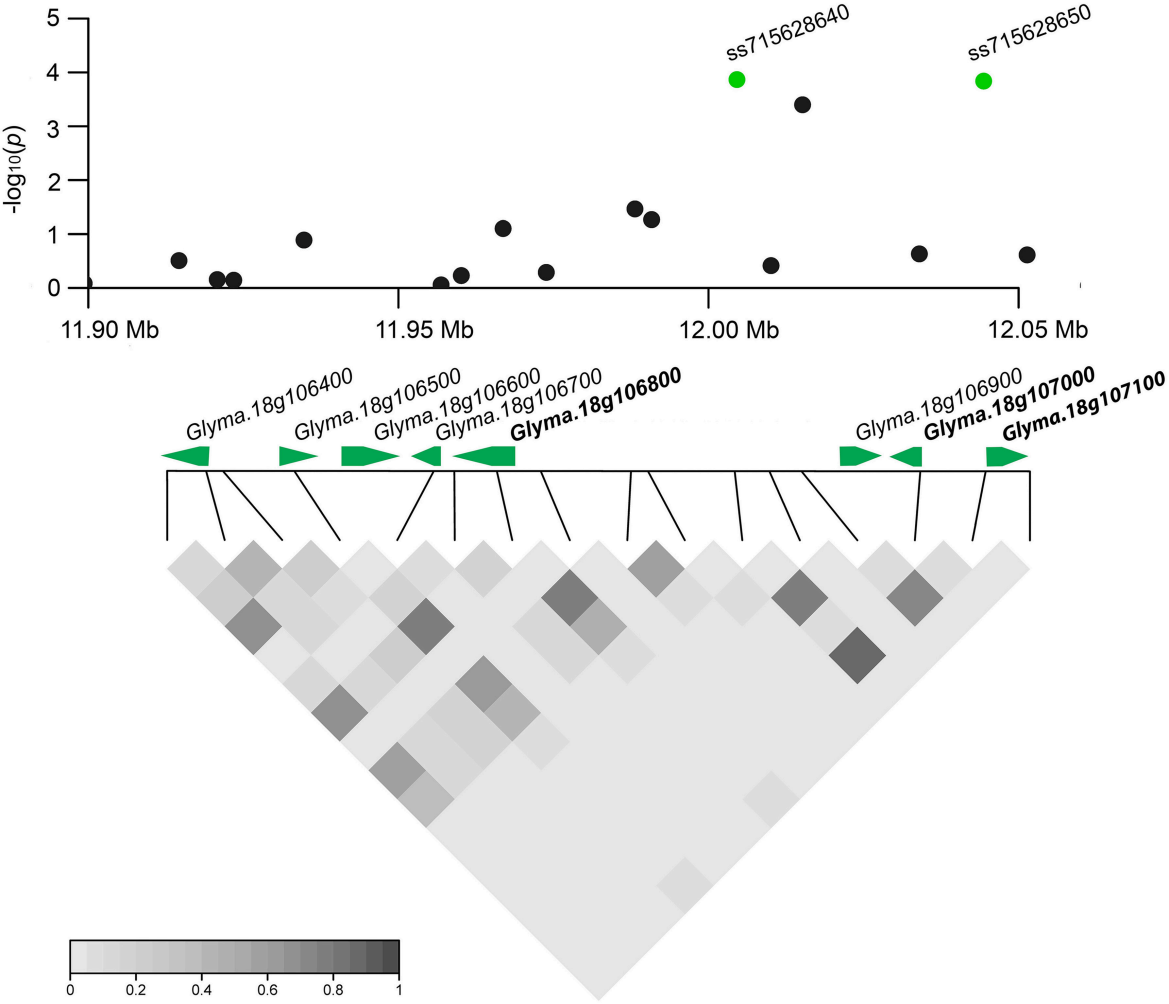
**Regional plot and candidate genes for loci ss715628640 and ss715628650**. The log10-tranformed *P*-values of two association SNPs and adjacent loci are plotted against physical positions on chromosome 18. The middle panel shows all predicted gene models in the region. The proposed candidate genes are in bold. The bottom panel indicates the extent of LD in the region based on pairwise *r*^2^-values. The *r*^2^-values in the LD triangles are indicated with a color intensity index.

Based on detailed annotations for the soybean reference genome in SoyBase (http://www.soybase.org), we further predicted candidate genes within the 50 kb region on each side of the 10 associated SNPs (Table 1). Only one (ss71561193) of the 10 SNPs is located within a gene, *Glyma.18g196200*, which encodes an mRNA capping enzyme family protein involved in transferring phosphorous-containing groups (SoyBase, http://www.soybase.org). The other nine SNPs are located in intergenic regions. A total of 58 gene models were predicted within the search region (Table S3), 16 of which are related to disease resistance based on published literature and considered to be candidate genes associated with SCN resistance (Table S4). This candidate list includes genes encoding leucine-rich repeat (LRR)-containing resistance (R) proteins or protein kinases (PKs), a receptor-like protein, a RING/U-box protein, and MYB family transcription factors. Briefly, two identified LRR-R genes (*Glyma.18g077900* and *Glyma.18g078000*) are close to SNP ss715632647. The gene *Glyma.18g078000*, which showed root-specific expression in soybean (Severin et al., 2010), is only 5.4 kb away from SNP ss715632647. In addition, two PK-encoding genes (*Glyma.18g106800, Glyma.18g107000*) and a phosphate acyltransferase-encoding gene, *Glyma.18g107100*, within a genomic region containing two tightly linked significant SNPs (ss715628640 and ss715628650) were identified as candidates (Figure 4). *Glyma.18g106800*, which encodes a mitogen-activated protein kinase (MAPK), is reported to be highly expressed in roots (Severin et al., 2010).

## Discussion

### Applying *G. soja* to improve SCN resistance

Wild crop relatives harbor novel and exotic genetic resources that are crucial for the development of insect pest management (Hajjar and Hodgkin, 2007). Some wild species have been applied in breeding practices, such as for tomato (Vidavski et al., 2008; Kumar et al., 2014). *G. soja* is widely distributed in China, Korea, Japan, and Northeast Russia, and long-term exposure to various environmental stresses in the wild has rendered *G. soja* able to adapt to diverse environments with severe abiotic and biotic stresses. Some of these beneficial traits, such as salt tolerance and pest resistance, may have been lost in cultivated soybean due to the effects of domestication “bottleneck.” Indeed, a recent study showed that the domestication bottleneck of soybean has resulted in more than a 50% reduction in diversity and the elimination of 81% of the rare alleles present in *G. soja* (Hyten et al., 2006). For example, Qi et al. (2014) recently identified a salt-tolerance gene, *GmCHX1*, unique to wild soybean. In addition, the limited availability of SCN-resistant soybean varieties and the selection for SCN populations that overcome existing resistance have been two major challenges to SCN management (Mitchum et al., 2007; Niblack et al., 2008). As an alternative, our study and several recent works (Winter et al., 2007; Kim et al., 2011; Kim and Diers, 2013) have identified resistant accessions among wild soybean populations, suggesting that wild soybean have great potential for improving SCN resistance. These newly identified resistant resources may contain both conserved loci and new resistance genes, as shown in our study (Table 1) and previous studies (Winter et al., 2007; Kim et al., 2011; Kim and Diers, 2013).

Conversely, the association panel used in this study has a broad geographical and ecological distribution in East Asia (Figure 1A), where the SCN populations most likely originated (Tylka and Marett, 2014). These untapped *G. soja* genetic resources are prime candidates for discovery of SCN resistance, and indeed, a large portion (3.4%) of the accessions in our *G. soja* population were identified SCN resistant. This result suggests that additional SCN-resistant accessions could be identified when more wild soybean accessions from broader ecological regions in East Asia are screened. Correlation of resistance level with geographic location revealed clear differences between accessions from Japan and South Korea compared to those from other locations, with the Japanese and South Korean accessions showing on average higher levels of resistance. This geographic resistance pattern was also observed in a recent study (Zheng et al., 2005) reporting 12 of 15 *Bean pod mottle virus*-tolerant *G. soja* genotypes from Japan. These differences might partially be caused by different selection pressures among regions. In fact, as the majority of resistant accessions were originally from Japan, wild soybean from Japan and South Korea might have experienced higher selection pressure from SCN HG 2.5.7 (Figure 1B). These results can serve as a guide for focussing on certain areas when not all samples can be screened.

Geographical origin is an important factor affecting population structure in soybean. Consistent with a recent study (Zhang et al., 2015a) focussing on a cultivated soybean population, we also found a high correlation between country of origin and population structure in the *G. soja* population (Figures 2C–E). It is clear that population structure was affected more by geographical origin than by mature group (MG), as despite belonging to different MGs (Figure 2C), the accessions within a country clustered together. A possible explanation for the mixture of accessions from China and Russia may be frequent material exchange or gene flow occurring in that area.

### Broad and novel genetic resources for SCN resistance

Development of soybean cultivars with broad resistance to diverse SCN HG types, as well as identification of novel resistant resources/genes, are urgently needed due to SCN HG type shifts caused by evolutionary arms races. The recent identification of two major loci, *rhg1* (Cook et al., 2012) and *Rhg4* (Liu et al., 2012), conferring SCN resistance (Concibido et al., 2004) has provided increasing knowledge and understanding about the genetic architecture and resistance mechanisms of soybean defenses against SCN. The genomic regions linked with *rhg1* are significantly associated with HG 2.5.7 resistance. *Rhg1*, one of the major loci conferring soybean resistance to multiple SCN HG types (previously called races; Concibido et al., 2004), has also been previously identified in a resistant *G. soja* accession (PI468916) by linkage mapping (Kim et al., 2011). Thus, it is not surprising that the *rhg1*-linked regions were also identified in our study significantly associated with HG 2.5.7 resistance. In addition, loci located at the opposite end of chromosome 18 as *rhg1* (Vuong et al., 2010; Kim et al., 2011; Kim and Diers, 2013; Bao et al., 2014) and on chromosome 19 (Vuong et al., 2015) identified in our study have also recently been suggested as contributing to broad SCN resistance. The consistent results observed in the present and previous studies suggest that the significant loci on chromosomes 18 and 19 might play important roles in conferring SCN resistance in both *G. max* and *G. soja* populations. These finding will significantly accelerate molecular marker development and SCN resistance breeding programmes (e.g., Kadam et al., 2016).

As major genes, such as *rhg1*, are valuable resources but often not durable (Kadam et al., 2016), novel sources of resistance and genes are needed for long-term SCN management. Most previous studies have focussed on a limited number of resistant soybean cultivars, whereas exploration of exotic sources and identification of novel genes are lagging. Additionally, different resistant genotypes often show varying genetic resistance mechanisms. For example, Peking-source *rhg1* requires *rhg4* for full function, though the widely used PI88788 source does not (e.g., Cook et al., 2012; Liu et al., 2012). Soybean accession PI 567516C is SCN resistant but lacks the two major genes *rhg1* and *rhg4* (Vuong et al., 2010). Previous studies on the different SCN-resistant accessions, such as PI90763, PI404166, Peking, and PI438439B, have indicated that their resistance to SCN HG 2.5.7 is controlled by different alleles at the same locus (Anand and Raoarelli, 1989). However, it is not yet clear whether the different levels of SCN resistance to HG 2.5.7 in the wild genetic resources, i.e., *G. soja* populations, are due to different alleles at the same locus or to different genes. Therefore, the identification of conserved and novel loci can further our understanding of the molecular mechanisms of the soybean-SCN interaction. It is clear that SCN resistance is a complex trait involving a number of genes that initiate a cascade of defense signaling and responses (Concibido et al., 2004). Overall, identification of these SCN-resistance loci in the non-domesticated *G. soja* population can improve our understanding of the genetic basis of SCN resistance and further accelerate the development of diverse soybean cultivars to mitigate damage caused by SCN.

### Candidate genes involved in SCN resistance

The known genes that confer resistance to SCN are unusual in that they do not encode canonical NB-LRR-type genes for resistance (detection) to other pathogens (DeYoung and Innes, 2006). The cloned *rhg1* (Cook et al., 2012) locus indicates that the copy number of three tandem genes (an amino acid transporter, an alpha-SNAP, and the WI12 protein) and *rhg-4* (Liu et al., 2012), a serine hydroxymethyltransferase, confer resistance to SCN. These known SCN resistance genes appear to function in metabolic or other plant cell processes, and it is unclear how they impart resistance to SCN, especially resistance that is specific to certain HG types. These known SCN resistance genes were not identified among the 58 gene models predicted in the *G. soja* search region analyzed in the present study.

Protein phosphorylation/dephosphorylation is one of the major mechanisms involved in plant-pathogen interactions (Xing and Laroche, 2011). The process of phosphorylation/dephosphorylation involves various types of protein kinases (PKs), such as calmodulin-dependent receptor-like protein kinases (CDPKs) and MAPKs. The involvement of various types of PKs in plant defense against nematodes has also been reported in other plant species. For example, it has been suggested that two *Arabidopsis* MAPK genes, *MPK3*, and *MPK6*, are important regulators of the plant-nematode interaction (Sidonskaya et al., 2016). In tobacco, a CDPK protein can trigger plant defense via signaling upon virus attack (Romeis et al., 2000), and in *Arabidopsis*, mutation of a receptor-like protein kinase (*RLK*) gene, *RPK2*, results in a decrease in both nematode infection and syncytium size in the *rpk2* mutant (Replogle et al., 2013). Such evidence strongly suggests that PKs are promising candidate genes involved in the soybean response to SCN attack. Regardless, involvement of MAPKs and CDPKs in the soybean-SCN interaction has not been comprehensively investigated because it remains challenging to pinpoint causal genes from ~200 MAPKs (Neupane et al., 2013) and ~500 receptor-like proteins (Zhou et al., 2016) in the soybean genome. As an alternative, our study identified one MAPK (*Glyma.18g106800*) and one CDPK (*Glyma.18g064100*) that might be involved in wild soybean defense against SCN; this result must be further verified.

Plant nucleotide-binding site-leucine-rich repeat (NBS-LRR) proteins have been extensively studied in plant defense against pathogens (DeYoung and Innes, 2006). Multiple LRR domains can recognize pathogen-encoded effector proteins, which trigger the defense response through a special signaling cascade (Jones and Jones, 1997). In *Arabidopsis*, RPM1, an intracellular innate immune receptor, can negatively control the extent of cell death and result in an overall resistance response at the site of infection (Boyes et al., 1998). A study of the *Pc* locus, which confers resistance to fungal pathogens, identified an NBS-LRR protein-encoding gene as the candidate gene (Nagy and Bennetzen, 2008). In addition, a significant change in NBS-LRR expression in roots was observed after SCN infection (Klink et al., 2007; Guo et al., 2015). Transcription factors such as RING and MYB might also be involved in signaling during soybean defense responses against SCN (Ithal et al., 2007; Hosseini and Matthews, 2014; Guo et al., 2015), and candidate RING, MYB, and NBS-LRR gene models were identified in the *G. soja* search region analyzed here.

A study by Guo et al. (2015) indicated that a soybean ortholog of the *Arabidopsis* gene *RPK2, GmRPK2B*, is strongly expressed in root tips, vascular tissues, and nematode feeding sites. Similarly, high or exclusive expression of genes encoding PKs (*Glyma.18g106800, Glyma.18g064100*) and LRR-R proteins (*Glyma.18g077900, Glyma.18g078000*) in roots (Severin et al., 2010) suggests their potential of playing specific roles in root defense. Future research should validate the functions of these candidate genes using root transformation assays.

## Conclusions and perspectives

In this study, 43 *G. soja* accessions showing resistance and moderate resistance to HG Type 2.5.7 were identified, suggesting the importance and great potential of *G. soja* as a novel and exotic genetic resource for SCN management. GWAS was successfully applied to dissect the genetic architecture of resistance to SCN HG Type 2.5.7 in the *G. soja* population. This study provides an example of exploring the untapped genetic resource of *G. soja* for novel gene identification, which may provide a rich source of alternative resistance for breeding SCN-resistant soybean cultivars. This work also highlighted the need and importance of utilizing wild resources for crop pest management.

## Author contributions

BS conceived the idea and acquired funding. HZ and BS designed the experiments. HZ and CL performed the experiments. HZ, JK, and JG performed the data analyses. JW assisted in the data visualization. HZ, BS, ED, and JG wrote the manuscript. All authors read and approved the final version of the manuscript for publication.

### Conflict of interest statement

The authors declare that the research was conducted in the absence of any commercial or financial relationships that could be construed as a potential conflict of interest.
