# Supplementary material for: Genome-Wide Association Study of Resistance to Soybean Cyst Nematode (Heterodera glycines) HG Type 2.5.7 in Wild Soybean (Glycine soja)
Source: Front Plant Sci. 2016 Aug 17;7:1214. doi: 10.3389/fpls.2016.01214 (PMC4987380; doi:10.3389/fpls.2016.01214)
Supplement: Supplementary file 1 [file Table1.DOCX]

Supplementary Material

Genome-Wide Association Analysis of Resistance to Soybean Cyst Nematode (Heterodera glycines) HG Type 2.5.7 in Wild Soybean (Glycine soja)

**Hengyou Zhang^1^, Chunying Li^2^, Eric L Davis^2^, Jinshe Wang^3^, Joshua D. Griffin^4^, Janice Kofsky^1^, and Bao-Hua Song^1^***

*** Correspondence:** Bao-Hua Song, bsong5@uncc.edu

# Supplementary Tables

**Table S1 Accession ID, country of origin, maturity group, and Female Index (FI) of the 235 *G soja* accessions used in this study. R, resistance. MR, moderate resistance. MS, moderate susceptible. S, susceptible.**

| **PI #** | **Mature Group** | **Country** | **Resistance level** | **Mean FI (%)** |
| --- | --- | --- | --- | --- |
| PI101404A | II | China | S | 64.9 |
| PI101404B | II | China | MR | 21.7 |
| PI163453 | VII | China | S | 98.4 |
| PI326582A | II | Russian Federation | MS | 52.3 |
| PI339732 | IV | Korea, South | S | 79.5 |
| PI339871A | V | Korea, South | MR | 28.0 |
| PI342621C | 0 | Russian Federation | S | 126.4 |
| PI366120 | IV | Japan | MR | 16.3 |
| PI366122 | IV | Japan | MR | 18.1 |
| PI366123 | IV | Japan | S | 61.8 |
| PI366124 | V | Japan | S | 62.0 |
| PI378683 | VI | Japan | S | 86.3 |
| PI378684A | VI | Japan | MR | 25.7 |
| PI378686A | VII | Japan | S | 86.8 |
| PI378690 | VII | Japan | S | 110.4 |
| PI378691 | VII | Japan | MS | 49.5 |
| PI378695A | VI | Japan | S | 72.4 |
| PI378697A | V | Japan | S | 122.6 |
| PI378698 | VI | Japan | MR | 21.5 |
| PI378699A | VII | Japan | MR | 20.6 |
| PI378701A | V | Japan | MS | 52.3 |
| PI406684 | III | Japan | S | 144.8 |
| PI407034 | V | Japan | S | 67.4 |
| PI407036 | V | Japan | S | 68.4 |
| PI407037 | V | Japan | MR | 26.4 |
| PI407038 | V | Japan | S | 123.3 |
| PI407044 | V | Japan | MS | 35.0 |
| PI407047 | V | Japan | S | 142.8 |
| PI407050 | V | Japan | S | 60.4 |
| PI407052 | V | Japan | MS | 39.6 |
| PI407053 | VI | Japan | S | 75.0 |
| PI407056 | VI | Japan | S | 196.7 |
| PI407077 | VII | Japan | S | 119.8 |
| PI407083 | VI | Japan | S | 108.1 |
| PI407089 | VI | Japan | S | 103.5 |
| PI407097 | VI | Japan | S | 95.1 |
| PI407120 | VII | Japan | S | 67.0 |
| PI407144 | VI | Japan | S | 227.4 |
| PI407157 | VI | Japan | S | 75.7 |
| PI407167 | V | Korea, South | MS | 52.4 |
| PI407174 | V | Korea, South | S | 138.4 |
| PI407175 | IV | Korea, South | MS | 36.8 |
| PI407183 | V | Korea, South | S | 110.1 |
| PI407184 | IV | Korea, South | S | 75.7 |
| PI407190 | V | Korea, South | S | 76.9 |
| PI407195 | IV | Korea, South | S | 86.6 |
| PI407198 | V | Korea, South | MS | 34.0 |
| PI407200 | IV | Korea, South | S | 150.3 |
| PI407201 | V | Korea, South | MS | 45.1 |
| PI407202 | V | Korea, South | S | 125.2 |
| PI407209 | IV | Korea, South | S | 106.3 |
| PI407217 | IV | Korea, South | MR | 29.7 |
| PI407220 | V | Korea, South | S | 71.5 |
| PI407221 | V | Korea, South | S | 77.4 |
| PI407229 | V | Korea, South | S | 111.5 |
| PI407235 | V | Korea, South | MS | 42.6 |
| PI407239 | V | Korea, South | MS | 34.0 |
| PI407240 | V | Korea, South | S | 65.3 |
| PI407241 | V | Korea, South | S | 55.9 |
| PI407246 | V | Korea, South | S | 61.1 |
| PI407248 | V | Korea, South | S | 63.4 |
| PI407249 | V | Korea, South | S | 72.0 |
| PI407254 | VI | Korea, South | MR | 29.4 |
| PI407262 | VI | Korea, South | MS | 46.3 |
| PI407267 | V | Korea, South | S | 83.0 |
| PI407270 | VI | Korea, South | S | 75.3 |
| PI407271 | V | Korea, South | S | 80.2 |
| PI407275 | IV | Korea, South | S | 104.2 |
| PI407278 | IV | Korea, South | S | 86.6 |
| PI407281 | VI | Korea, South | MS | 47.5 |
| PI407286 | VI | Japan | MS | 41.7 |
| PI407288 | II | China | S | 89.2 |
| PI407296 | II | China | S | 138.4 |
| PI407298 | II | China | S | 63.9 |
| PI407302 | V | China | S | 69.0 |
| PI407304 | VI | China | MS | 42.7 |
| PI407307 | VI | China | S | 75.0 |
| PI407308 | V | Korea, South | MR | 26.9 |
| PI407315 | V | Korea, South | S | 100.2 |
| PI407318A | V | Korea, South | S | 87.2 |
| PI407322 | V | Korea, South | MS | 53.7 |
| PI423990A | 0 | Russian Federation | S | 141.4 |
| PI423993 | 0 | Russian Federation | MS | 34.4 |
| PI423995 | 0 | Russian Federation | S | 131.3 |
| PI423997 | 0 | Russian Federation | S | 92.0 |
| PI423999B | 0 | Russian Federation | MS | 52.8 |
| PI424000 | 0 | Russian Federation | S | 63.9 |
| PI424001 | 0 | Russian Federation | S | 119.2 |
| PI424002 | 0 | Russian Federation | MR | 11.8 |
| PI424008A | V | Korea, South | S | 93.1 |
| PI424014 | V | Korea, South | S | 90.6 |
| PI424025B | V | Korea, South | S | 79.9 |
| PI424032 | IV | Korea, South | S | 90.0 |
| PI424059B | V | Korea, South | MS | 54.4 |
| PI424063 | IV | Korea, South | S | 85.9 |
| PI424064 | V | Korea, South | S | 88.9 |
| PI424079 | IV | Korea, South | S | 109.0 |
| PI424082 | V | Korea, South | MS | 38.9 |
| PI424088 | IV | Korea, South | S | 58.9 |
| PI424091A | IV | Korea, South | S | 60.4 |
| PI424093 | V | Korea, South | R | 5.2 |
| PI424096 | V | Korea, South | MR | 14.1 |
| PI424102A | V | Korea, South | S | 61.6 |
| PI424117 | V | Korea, South | MR | 25.7 |
| PI424121 | V | Korea, South | MS | 49.0 |
| PI447003A | 0 | China | MR | 21.7 |
| PI458537A | 0 | China | S | 81.3 |
| PI458540D | 0 | China | S | 139.2 |
| PI464866A | 0 | China | MS | 44.1 |
| PI464889C | II | China | S | 77.8 |
| PI464890A | II | China | MS | 34.4 |
| PI464891B | II | China | MS | 43.6 |
| PI464925C | I | China | MS | 42.7 |
| PI464926 | 0 | China | S | 88.2 |
| PI464927A | 0 | China | S | 84.7 |
| PI464927B | 0 | China | MR | 11.5 |
| PI464928 | 0 | China | S | 112.8 |
| PI464936B | VI | China | R | 9.0 |
| PI464937A | VI | China | R | 5.7 |
| PI468396B | IV | China | S | 149.0 |
| PI468397A | IV | China | S | 137.5 |
| PI468398B | IV | China | S | 81.0 |
| PI468399B | IV | China | S | 62.0 |
| PI468918 | III | China | MR | 19.1 |
| PI479745 | I | China | S | 87.0 |
| PI479746B | II | China | MS | 39.8 |
| PI479749 | III | China | S | 95.1 |
| PI479750 | I | China | MS | 34.3 |
| PI479751 | III | China | MS | 30.7 |
| PI483466 | V | China | MS | 38.5 |
| PI483468A | V | China | S | 82.6 |
| PI486220 | IX | Japan | NA | NA |
| PI487428 | V | Japan | R | 9.0 |
| PI487430 | V | Japan | MS | 31.5 |
| PI487431 | IX | Japan | MR | 28.5 |
| PI504287A | IV | Japan | S | 98.6 |
| PI507582 | V | Japan | S | 69.9 |
| PI507595 | VI | Japan | S | 94.1 |
| PI507609 | VI | Japan | MS | 40.7 |
| PI507615 | VI | Japan | R | 8.3 |
| PI507619B | VI | Japan | MS | 56.3 |
| PI507632 | VII | Japan | S | 61.3 |
| PI507638 | VI | Japan | S | 82.5 |
| PI507644 | VI | Japan | S | 84.4 |
| PI507656 | VII | Japan | S | 72.2 |
| PI507667 | VI | Japan | R | 9.9 |
| PI507722 | 0 | Russian Federation | MS | 49.0 |
| PI507723B | II | Russian Federation | S | 95.5 |
| PI507725B | 0 | Russian Federation | MS | 57.6 |
| PI507727 | 0 | Russian Federation | S | 110.9 |
| PI507729 | 0 | Russian Federation | S | 137.0 |
| PI507730 | 0 | Russian Federation | S | 179.5 |
| PI507731 | 0 | Russian Federation | S | 72.9 |
| PI507734 | 0 | Russian Federation | S | 69.4 |
| PI507735 | 0 | Russian Federation | MS | 48.3 |
| PI507738 | 0 | Russian Federation | MR | 26.6 |
| PI507739B | 0 | Russian Federation | MR | 17.5 |
| PI507740 | 0 | Russian Federation | MS | 38.4 |
| PI507742 | 0 | Russian Federation | S | 103.5 |
| PI507749 | 0 | Russian Federation | S | 94.0 |
| PI507752 | 0 | Russian Federation | S | 134.2 |
| PI507757 | 0 | Russian Federation | S | 75.9 |
| PI507759 | 0 | Russian Federation | MS | 35.6 |
| PI507760 | 0 | Russian Federation | MS | 51.4 |
| PI507764 | 0 | Russian Federation | MS | 38.4 |
| PI507774 | II | Russian Federation | MS | 39.4 |
| PI507776 | I | Russian Federation | MS | 30.0 |
| PI507777 | I | Russian Federation | S | 79.3 |
| PI507782 | 0 | Russian Federation | MS | 32.8 |
| PI507784 | II | Russian Federation | S | 116.8 |
| PI507787 | II | Russian Federation | MS | 39.6 |
| PI507788 | III | Russian Federation | S | 128.1 |
| PI507794 | I | Russian Federation | MS | 54.7 |
| PI507798 | II | Russian Federation | MS | 34.3 |
| PI507799 | I | Russian Federation | S | 57.1 |
| PI507803 | 0 | Russian Federation | S | 79.2 |
| PI507805 | 0 | Russian Federation | MR | 27.6 |
| PI507806A | 0 | Russian Federation | S | 72.7 |
| PI507814 | 0 | Russian Federation | MS | 43.1 |
| PI507816 | 0 | Russian Federation | S | 146.5 |
| PI507818B | 0 | Russian Federation | S | 86.8 |
| PI507821 | 0 | Russian Federation | MS | 57.6 |
| PI507826 | 0 | Russian Federation | S | 126.7 |
| PI507830B | 0 | Russian Federation | S | 138.4 |
| PI507833 | 0 | Russian Federation | S | 84.4 |
| PI507836 | 0 | Russian Federation | MS | 44.3 |
| PI507839 | 0 | Russian Federation | MR | 24.3 |
| PI507847 | II | Russian Federation | S | 88.2 |
| PI508066 | IV | Japan | S | 94.9 |
| PI508067 | IV | Japan | R | 5.8 |
| PI508069 | IV | Japan | S | 78.5 |
| PI514674 | III | Japan | S | 66.2 |
| PI522179 | 0 | China | MR | 19.4 |
| PI522180 | 0 | China | MR | 27.1 |
| PI522182A | 0 | China | MS | 52.1 |
| PI522182B | I | China | S | 61.1 |
| PI522193 | 0 | Russian Federation | MR | 26.0 |
| PI522196A | 0 | Russian Federation | MR | 28.8 |
| PI522198A | I | Russian Federation | MR | 27.6 |
| PI522200A | II | Russian Federation | MR | 20.0 |
| PI522211B | III | Russian Federation | S | 71.2 |
| PI522217 | II | Russian Federation | S | 63.2 |
| PI522223 | II | Russian Federation | MR | 27.1 |
| PI522226 | 0 | Russian Federation | S | 63.9 |
| PI522227 | II | Russian Federation | MS | 50.0 |
| PI522229 | 0 | Russian Federation | MR | 29.9 |
| PI522230A | 0 | Russian Federation | S | 62.8 |
| PI522234 | I | Russian Federation | MS | 38.2 |
| PI522235A | I | Russian Federation | MS | 37.7 |
| PI532450 | I | China | MS | 42.7 |
| PI532453A | III | China | MR | 24.5 |
| PI549037 | III | China | MR | 19.1 |
| PI549046 | IV | China | S | 100.3 |
| PI549047 | III | China | S | 66.5 |
| PI549048 | III | China | S | 67.2 |
| PI562531 | Unkown | Korea, South | MS | 30.6 |
| PI562534 | Unkown | Korea, South | MR | 28.7 |
| PI562544 | Unkown | Korea, South | MS | 36.5 |
| PI562550 | Unkown | Korea, South | MR | 16.8 |
| PI562551 | Unkown | Korea, South | S | 75.5 |
| PI562556 | Unkown | Korea, South | S | 77.5 |
| PI562557 | Unkown | Korea, South | S | 85.4 |
| PI562558 | Unkown | Korea, South | S | 103.2 |
| PI562565 | Unkown | Korea, South | MS | 35.2 |
| PI562568 | Unkown | Korea, South | S | 65.7 |
| PI578336 | Unkown | Russian Federation | S | 95.5 |
| PI578338A | Unkown | Russian Federation | MR | 24.1 |
| PI578345 | Unkown | Russian Federation | R | 3.3 |
| PI578353A | Unkown | Russian Federation | MS | 48.6 |
| PI597458B | Unkown | China | S | 81.3 |
| PI597459C | Unkown | China | S | 99.8 |
| PI597461B | Unkown | China | MS | 39.9 |
| PI597471D | Unkown | China | S | 111.3 |
| PI639590A | Unkown | Russian Federation | MS | 58.0 |
| PI639621 | Unkown | Russian Federation | S | 140.3 |

**Table S2. HG type 2.5.7 determination using seven soybean indicator lines.**

|  | **Entry** | **Rep 1** | **Rep 2** | **Rep 3** | **Rep 4** | **Mean** | **Female Index** | **Rating***** |
| --- | --- | --- | --- | --- | --- | --- | --- | --- |
| indicator line 1 = Peking | **Peking** | 2 | 3 | 0 | 2 | 1.8 | 3.4 | R |
| indicator line 2 = PI88788 | **PI 88788** | 33 | 11 | 18 | 6 | 17.0 | **33.2** | MS |
| indicator line 3 = PI90763 | **PI 90763** | 0 | 0 | 1 | 1 | 0.5 | 1.0 | R |
| indicator line 4 = PI437654 | **PI 437654** | 0 | 0 | 3 | 0 | 0.8 | 1.5 | R |
| indicator line 5 = PI209332 | **PI 209332** | 87 | 109 | 68 | 30 | 73.5 | **143.7** | S |
| indicator line 6 = PI89772 | **PI 89772** | 0 | 2 | 2 | 1 | 1.3 | 2.4 | R |
| indicator line 7 = PI 548316 | **PI 548316** | 70 | 54 | 55 | 3 | 45.5 | **89.0** | S |
|  | **Williams 82** | 41 | 86 | 84 | 28 | 59.8 | 116.8 | S |

Table S3 All 58 genes that were identified within 100kb region of the significant SNP.

| SNPs | LocusName | Chr | Start | End | Best-hit-arabi-name | Arabi-symbol | Arabi-defline |
| --- | --- | --- | --- | --- | --- | --- | --- |
| ss715631923 | Glyma.18G063000 | Chr18 | 5,762,616 | 5,768,784 | AT5G58720.1 |  | smr (Small MutS Related) domain-containing protein |
|  | Glyma.18G063100 | Chr18 | 5,772,057 | 5,774,497 | AT5G58740.1 |  | HSP20-like chaperones superfamily protein |
|  | Glyma.18G063200 | Chr18 | 5,779,244 | 5,784,529 | AT2G38170.3 | ATCAX1,CAX1,RCI4 | cation exchanger 1 |
|  | Glyma.18G063300 | Chr18 | 5,788,627 | 5,790,510 | AT5G58750.1 |  | NAD(P)-binding Rossmann-fold superfamily protein |
|  | Glyma.18G063400 | Chr18 | 5,796,259 | 5,805,903 | AT2G29940.1 | ATPDR3,PDR3 | pleiotropic drug resistance 3 |
|  | Glyma.18G063500 | Chr18 | 5,828,968 | 5,831,963 | AT3G47160.1 |  | RING/U-box superfamily protein |
|  | Glyma.18G063600 | Chr18 | 5,834,557 | 5,834,989 |  |  |  |
|  | Glyma.18G063700 | Chr18 | 5,836,067 | 5,836,651 |  |  |  |
|  | Glyma.18G063800 | Chr18 | 5,842,344 | 5,842,744 | AT1G11655.1 |  |  |
|  | Glyma.18G063900 | Chr18 | 5,843,844 | 5,845,158 |  |  |  |
|  | Glyma.18G064000 | Chr18 | 5,847,976 | 5,851,846 | AT1G07230.1 | NPC1 | non-specific phospholipase C1 |
|  | Glyma.18G064100 | Chr18 | 5,853,137 | 5,857,011 | AT5G58940.1 | CRCK1 | calmodulin-binding receptor-like cytoplasmic kinase 1 |
|  | Glyma.18G064200 | Chr18 | 5,863,321 | 5,864,449 | AT1G07230.1 | NPC1 | non-specific phospholipase C1 |
| ss715632647 | Glyma.18G077800 | Chr18 | 7,411,806 | 7,413,449 | AT2G04480.1 |  |  |
|  | Glyma.18G077900 | Chr18 | 7,420,049 | 7,421,741 | AT1G53350.1 |  | Disease resistance protein (CC-NBS-LRR class) family |
|  | Glyma.18G078000 | Chr18 | 7,441,703 | 7,445,003 | AT3G07040.1 | RPM1,RPS3 | NB-ARC domain-containing disease resistance protein |
|  | Glyma.18G078100 | Chr18 | 7,456,993 | 7,459,469 | AT4G16400.1 |  | hypothetical protein GLYMA_18G078100 |
|  | Glyma.18G078200 | Chr18 | 7,483,760 | 7,484,878 | AT3G13130.1 |  | neurofilament light polypetide-like |
| ss715628640 | Glyma.18G106700 | Chr18 | 11,956,201 | 11,957,138 | AT3G58840.1 |  | Tropomyosin-related |
| ss715628650 | Glyma.18G106800 | Chr18 | 11,959,920 | 11,966,545 | AT2G42880.1 | ATMPK20,MPK20 | MAP kinase 20 |
|  | Glyma.18G106900 | Chr18 | 12,018,996 | 12,026,786 | AT1G29400.1 | AML5,ML5 | MEI2-like protein 5 |
|  | Glyma.18G107000 | Chr18 | 12,028,945 | 12,032,564 | AT2G42910.1 |  | Phosphoribosyltransferase family protein |
|  | Glyma.18G107100 | Chr18 | 12,047,167 | 12,050,021 | AT1G06520.1 | ATGPAT1,GPAT1 | glycerol-3-phosphate acyltransferase 1 |
|  | Glyma.18G107200 | Chr18 | 12,059,922 | 12,060,860 | AT2G42900.1 |  | Plant basic secretory protein (BSP) family protein |
|  | Glyma.18G107300 | Chr18 | 12,091,200 | 12,095,291 | AT2G30933.1 |  | Carbohydrate-binding X8 domain superfamily protein |
| ss715631131 | Glyma.18G193400 | Chr18 | 46,622,868 | 46,628,926 | AT5G01050.1 |  | Laccase/Diphenol oxidase family protein |
|  | Glyma.18G193500 | Chr18 | 46,624,396 | 46,624,602 |  |  |  |
|  | Glyma.18G193600 | Chr18 | 46,669,124 | 46,671,854 | AT3G54050.2 | HCEF1 | high cyclic electron flow 1 |
|  | Glyma.18G193700 | Chr18 | 46,672,107 | 46,675,595 | AT2G37960.2 |  |  |
|  | Glyma.18G193800 | Chr18 | 46,693,001 | 46,696,315 | AT5G49290.1 | ATRLP56,RLP56 | receptor like protein 56 |
| ss715631193 | Glyma.18G196000 | Chr18 | 47,086,481 | 47,093,157 | AT2G38040.2 | CAC3 | acetyl Co-enzyme a carboxylase carboxyltransferase alpha subunit |
|  | Glyma.18G196100 | Chr18 | 47,089,797 | 47,090,081 |  |  |  |
|  | Glyma.18G196200 | Chr18 | 47,102,746 | 47,129,707 | AT3G09100.2 |  | mRNA capping enzyme family protein |
|  | Glyma.18G196300 | Chr18 | 47,132,249 | 47,134,819 | AT3G09085.1 |  | Protein of unknown function (DUF962) |
|  | Glyma.18G196400 | Chr18 | 47,133,514 | 47,133,826 |  |  |  |
| ss715631383 | Glyma.18G203300 | Chr18 | 48,457,541 | 48,462,209 | AT3G08950.1 |  | electron transport SCO1/SenC family protein |
|  | Glyma.18G203400 | Chr18 | 48,465,473 | 48,472,683 | AT3G51820.1 | ATG4,CHLG,G4 | UbiA prenyltransferase family protein |
|  | Glyma.18G203500 | Chr18 | 48,472,956 | 48,473,972 | AT3G51810.1 | AT3,ATEM1,EM1,GEA1 | Stress induced protein |
|  | Glyma.18G203600 | Chr18 | 48,513,938 | 48,517,959 |  |  |  |
| ss715631522 | Glyma.18G210300 | Chr18 | 49,597,315 | 49,611,969 | AT3G08850.1 | ATRAPTOR1B,RAPTOR1,RAPTOR1B | HEAT repeat ;WD domain, G-beta repeat protein protein |
|  | Glyma.18G210400 | Chr18 | 49,623,839 | 49,631,767 | AT4G14368.1 |  | Regulator of chromosome condensation (RCC1) family protein |
|  | Glyma.18G210500 | Chr18 | 49,642,348 | 49,649,886 | AT3G08860.1 | PYD4 | PYRIMIDINE 4 |
|  | Glyma.18G210600 | Chr18 | 49,690,288 | 49,692,778 | AT3G51710.1 |  | D-mannose binding lectin protein with Apple-like carbohydrate-binding domain |
| ss715634285 | Glyma.19G105400 | Chr19 | 35,541,994 | 35,545,213 | AT5G13920.1 |  | GRF zinc finger / Zinc knuckle protein |
|  | Glyma.19G105500 | Chr19 | 35,568,016 | 35,571,441 | AT5G13920.1 |  | GRF zinc finger / Zinc knuckle protein |
|  | Glyma.19G105600 | Chr19 | 35,572,057 | 35,575,895 | AT5G51050.1 |  | Mitochondrial substrate carrier family protein |
|  | Glyma.19G105700 | Chr19 | 35,614,441 | 35,616,086 | AT3G61110.1 | ARS27A,RS27A | ribosomal protein S27 |
| ss715634622 | Glyma.19G119200 | Chr19 | 37,677,773 | 37,683,394 | AT3G03630.1 | CS26 | cysteine synthase 26 |
|  | Glyma.19G119300 | Chr19 | 37,702,506 | 37,704,532 | AT1G08810.1 | AtMYB60,MYB60 | myb domain protein 60 |
|  | Glyma.19G119400 | Chr19 | 37,709,968 | 37,712,608 | AT1G08710.1 |  | F-box family protein |
|  | Glyma.19G119500 | Chr19 | 37,713,894 | 37,721,619 |  |  |  |
|  | Glyma.19G119600 | Chr19 | 37,714,236 | 37,715,659 |  |  |  |
|  | Glyma.19G119700 | Chr19 | 37,722,073 | 37,722,635 |  |  |  |
|  | Glyma.19G119800 | Chr19 | 37,722,659 | 37,724,669 | AT1G08700.1 | PS1 | Presenilin-1 |
|  | Glyma.19G119900 | Chr19 | 37,726,130 | 37,726,533 |  |  |  |
|  | Glyma.19G120000 | Chr19 | 37,727,145 | 37,731,532 | AT3G26430.1 |  | GDSL-like Lipase/Acylhydrolase superfamily protein |
|  | Glyma.19G120100 | Chr19 | 37,759,477 | 37,764,533 | AT5G17710.2 | EMB1241 | Co-chaperone GrpE family protein |
|  | Glyma.19G120200 | Chr19 | 37,773,951 | 37,778,723 | AT1G33110.1 |  | MATE efflux family protein |

Table S4 Levels of Linkage disequilibrium (LD) within 100 kb region (50 kb adjacent to each side of the significant SNP)

| Significant SNPs | Significant SNPs_Position | Locus1 | Locus1_position | Locus1_variants | Locus2 | Locus2_position | Locus2_variants | Dist_bp | R^2 | DPrime | pDiseq |
| --- | --- | --- | --- | --- | --- | --- | --- | --- | --- | --- | --- |
| ss715631923 | 5,814,672 | Chr18 | 5,798,918 | G:A | Chr18 | 5,742,223 | G:A | 56,695 | 0.035364 | 0.398438 | 0.01416 |
|  |  | Chr18 | 5,814,672 | G:A | Chr18 | 5,742,223 | G:A | 72,449 | 0.005645 | 0.088041 | 0.28719 |
|  |  | Chr18 | 5,814,672 | G:A | Chr18 | 5,798,918 | G:A | 15,754 | 0.150039 | 0.743937 | 1.08E-05 |
|  |  | Chr18 | 5,825,023 | T:C | Chr18 | 5,742,223 | G:A | 82,800 | 0.001761 | 0.104776 | 0.70717 |
|  |  | Chr18 | 5,825,023 | T:C | Chr18 | 5,798,918 | G:A | 26,105 | 0.009921 | 0.336937 | 0.19826 |
|  |  | Chr18 | 5,825,023 | T:C | Chr18 | 5,814,672 | G:A | 10,351 | 0.011786 | 0.304517 | 0.2129 |
|  |  | Chr18 | 5,881,160 | A:G | Chr18 | 5,798,918 | G:A | 82,242 | 0.057305 | 0.426768 | 0.00388 |
|  |  | Chr18 | 5,881,160 | A:G | Chr18 | 5,814,672 | G:A | 66,488 | 1.01E-05 | 0.003233 | 1 |
|  |  | Chr18 | 5,881,160 | A:G | Chr18 | 5,825,023 | T:C | 56,137 | 0.009676 | 0.201455 | 0.19455 |
|  |  |  |  |  |  |  |  |  |  |  |  |
| ss715632647 | 7,450,433 | Chr18 | 7,423,285 | A:G | Chr18 | 7,403,773 | G:A | 19,512 | 0.029154 | 0.17716 | 0.0145 |
|  |  | Chr18 | 7,433,942 | G:A | Chr18 | 7,403,773 | G:A | 30,169 | 0.018409 | 0.357143 | 0.06737 |
|  |  | Chr18 | 7,433,942 | G:A | Chr18 | 7,423,285 | A:G | 10,657 | 0.127831 | 0.872829 | 1.89E-08 |
|  |  | Chr18 | 7,436,958 | G:A | Chr18 | 7,403,773 | G:A | 33,185 | 0.001709 | 0.345238 | 0.6165 |
|  |  | Chr18 | 7,436,958 | G:A | Chr18 | 7,423,285 | A:G | 13,673 | 0.002074 | 0.369748 | 0.6027 |
|  |  | Chr18 | 7,436,958 | G:A | Chr18 | 7,433,942 | G:A | 3,016 | 0.001964 | 1 | 1 |
|  |  | Chr18 | 7,446,140 | G:A | Chr18 | 7,403,773 | G:A | 42,367 | 0.008048 | 0.596364 | 0.36941 |
|  |  | Chr18 | 7,446,140 | G:A | Chr18 | 7,423,285 | A:G | 22,855 | 0.006867 | 0.57963 | 0.37242 |
|  |  | Chr18 | 7,446,140 | G:A | Chr18 | 7,433,942 | G:A | 12,198 | 0.003241 | 1 | 1 |
|  |  | Chr18 | 7,446,140 | G:A | Chr18 | 7,436,958 | G:A | 9,182 | 0.060363 | 0.318584 | 0.06381 |
|  |  | Chr18 | 7,450,433 | C:T | Chr18 | 7,403,773 | G:A | 46,660 | 0.014073 | 0.441441 | 0.10017 |
|  |  | Chr18 | 7,450,433 | C:T | Chr18 | 7,423,285 | A:G | 27,148 | 0.042893 | 0.848299 | 0.00168 |
|  |  | Chr18 | 7,450,433 | C:T | Chr18 | 7,433,942 | G:A | 16,491 | 7.28E-05 | 0.012987 | 1 |
|  |  | Chr18 | 7,450,433 | C:T | Chr18 | 7,436,958 | G:A | 13,475 | 8.88E-04 | 1 | 1 |
|  |  | Chr18 | 7,450,433 | C:T | Chr18 | 7,446,140 | G:A | 4,293 | 0.044602 | 0.360748 | 0.03157 |
|  |  | Chr18 | 7,466,574 | G:N | Chr18 | 7,403,773 | G:A | 62,801 | NaN | NaN | NaN |
|  |  | Chr18 | 7,466,574 | G:N | Chr18 | 7,423,285 | A:G | 43,289 | NaN | NaN | NaN |
|  |  | Chr18 | 7,466,574 | G:N | Chr18 | 7,433,942 | G:A | 32,632 | NaN | NaN | NaN |
|  |  | Chr18 | 7,466,574 | G:N | Chr18 | 7,436,958 | G:A | 29,616 | NaN | NaN | NaN |
|  |  | Chr18 | 7,466,574 | G:N | Chr18 | 7,446,140 | G:A | 20,434 | NaN | NaN | NaN |
|  |  | Chr18 | 7,466,574 | G:N | Chr18 | 7,450,433 | C:T | 16,141 | NaN | NaN | NaN |
|  |  | Chr18 | 7,472,703 | A:G | Chr18 | 7,403,773 | G:A | 68,930 | 0.021001 | 0.473684 | 0.0525 |
|  |  | Chr18 | 7,472,703 | A:G | Chr18 | 7,423,285 | A:G | 49,418 | 0.005704 | 0.219633 | 0.27099 |
|  |  | Chr18 | 7,472,703 | A:G | Chr18 | 7,433,942 | G:A | 38,761 | 0.001291 | 0.290909 | 0.74803 |
|  |  | Chr18 | 7,472,703 | A:G | Chr18 | 7,436,958 | G:A | 35,745 | 0.052778 | 0.633333 | 0.0224 |
|  |  | Chr18 | 7,472,703 | A:G | Chr18 | 7,446,140 | G:A | 26,563 | 0.002287 | 1 | 1 |
|  |  | Chr18 | 7,472,703 | A:G | Chr18 | 7,450,433 | C:T | 22,270 | 0.005947 | 1 | 0.6135 |
|  |  | Chr18 | 7,472,703 | A:G | Chr18 | 7,466,574 | G:N | 6,129 | NaN | NaN | NaN |
|  |  | Chr18 | 7,477,007 | G:A | Chr18 | 7,403,773 | G:A | 73,234 | 9.18E-04 | 0.075152 | 0.69754 |
|  |  | Chr18 | 7,477,007 | G:A | Chr18 | 7,423,285 | A:G | 53,722 | 0.001075 | 0.080882 | 0.71062 |
|  |  | Chr18 | 7,477,007 | G:A | Chr18 | 7,433,942 | G:A | 43,065 | 2.73E-04 | 0.101961 | 1 |
|  |  | Chr18 | 7,477,007 | G:A | Chr18 | 7,436,958 | G:A | 40,049 | 0.002359 | 1 | 1 |
|  |  | Chr18 | 7,477,007 | G:A | Chr18 | 7,446,140 | G:A | 30,867 | 0.003892 | 1 | 1 |
|  |  | Chr18 | 7,477,007 | G:A | Chr18 | 7,450,433 | C:T | 26,574 | 0.062961 | 0.410995 | 0.00156 |
|  |  | Chr18 | 7,477,007 | G:A | Chr18 | 7,466,574 | G:N | 10,433 | NaN | NaN | NaN |
|  |  | Chr18 | 7,477,007 | G:A | Chr18 | 7,472,703 | A:G | 4,304 | 0.018531 | 1 | 0.05189 |
|  |  | Chr18 | 7,486,454 | T:G | Chr18 | 7,403,773 | G:A | 82,681 | 0.007487 | 0.2 | 0.26901 |
|  |  | Chr18 | 7,486,454 | T:G | Chr18 | 7,423,285 | A:G | 63,169 | 0.007858 | 0.190009 | 0.21427 |
|  |  | Chr18 | 7,486,454 | T:G | Chr18 | 7,433,942 | G:A | 52,512 | 7.17E-04 | 0.16036 | 0.79527 |
|  |  | Chr18 | 7,486,454 | T:G | Chr18 | 7,436,958 | G:A | 49,496 | 0.025093 | 0.603093 | 0.06755 |
|  |  | Chr18 | 7,486,454 | T:G | Chr18 | 7,446,140 | G:A | 40,314 | 0.009018 | 0.283077 | 0.18792 |
|  |  | Chr18 | 7,486,454 | T:G | Chr18 | 7,450,433 | C:T | 36,021 | 5.71E-05 | 0.069388 | 1 |
|  |  | Chr18 | 7,486,454 | T:G | Chr18 | 7,466,574 | G:N | 19,880 | NaN | NaN | NaN |
|  |  | Chr18 | 7,486,454 | T:G | Chr18 | 7,472,703 | A:G | 13,751 | 0.387769 | 0.837894 | 5.74E-15 |
|  |  | Chr18 | 7,486,454 | T:G | Chr18 | 7,477,007 | G:A | 9,447 | 0.034332 | 1 | 0.002 |
|  |  |  |  |  |  |  |  |  |  |  |  |
| ss715628640 | 12,004,584 | Chr18 | 11,956,877 | C:A | Chr18 | 11,934,773 | C:T | 22,104 | 0.047023 | 0.882883 | 3.31E-04 |
| ss715628650 | 12,044,370 | Chr18 | 11,960,134 | G:T | Chr18 | 11,934,773 | C:T | 25,361 | 0.177566 | 0.874444 | 5.62E-12 |
|  |  | Chr18 | 11,960,134 | G:T | Chr18 | 11,956,877 | C:A | 3,257 | 0.058824 | 1 | 4.83E-05 |
|  |  | Chr18 | 11,966,880 | G:A | Chr18 | 11,934,773 | C:T | 32,107 | 0.776249 | 0.916919 | 4.62E-44 |
|  |  | Chr18 | 11,966,880 | G:A | Chr18 | 11,956,877 | C:A | 10,003 | 0.054537 | 1 | 5.97E-05 |
|  |  | Chr18 | 11,966,880 | G:A | Chr18 | 11,960,134 | G:T | 6,746 | 0.194006 | 0.955366 | 2.11E-13 |
|  |  | Chr18 | 11,973,840 | C:T | Chr18 | 11,934,773 | C:T | 39,067 | 0.045067 | 1 | 3.20E-04 |
|  |  | Chr18 | 11,973,840 | C:T | Chr18 | 11,956,877 | C:A | 16,963 | 0.012545 | 1 | 0.14064 |
|  |  | Chr18 | 11,973,840 | C:T | Chr18 | 11,960,134 | G:T | 13,706 | 0.013467 | 0.535417 | 0.13024 |
|  |  | Chr18 | 11,973,840 | C:T | Chr18 | 11,966,880 | G:A | 6,960 | 0.042528 | 1 | 6.08E-04 |
|  |  | Chr18 | 11,988,139 | T:G | Chr18 | 11,934,773 | C:T | 53,366 | 0.626244 | 0.799259 | 1.96E-34 |
|  |  | Chr18 | 11,988,139 | T:G | Chr18 | 11,956,877 | C:A | 31,262 | 0.047602 | 0.883383 | 3.22E-04 |
|  |  | Chr18 | 11,988,139 | T:G | Chr18 | 11,960,134 | G:T | 28,005 | 0.141803 | 0.78691 | 2.43E-09 |
|  |  | Chr18 | 11,988,139 | T:G | Chr18 | 11,966,880 | G:A | 21,259 | 0.788822 | 0.915778 | 4.48E-44 |
|  |  | Chr18 | 11,988,139 | T:G | Chr18 | 11,973,840 | C:T | 14,299 | 0.045177 | 1 | 6.03E-04 |
|  |  | Chr18 | 11,990,829 | C:T | Chr18 | 11,934,773 | C:T | 56,056 | 0.420013 | 0.794412 | 3.53E-24 |
|  |  | Chr18 | 11,990,829 | C:T | Chr18 | 11,956,877 | C:A | 33,952 | 0.010874 | 0.349359 | 0.14024 |
|  |  | Chr18 | 11,990,829 | C:T | Chr18 | 11,960,134 | G:T | 30,695 | 0.119689 | 0.597243 | 1.20E-07 |
|  |  | Chr18 | 11,990,829 | C:T | Chr18 | 11,966,880 | G:A | 23,949 | 0.50951 | 0.903599 | 9.22E-30 |
|  |  | Chr18 | 11,990,829 | C:T | Chr18 | 11,973,840 | C:T | 16,989 | 0.050705 | 0.87766 | 4.90E-04 |
|  |  | Chr18 | 11,990,829 | C:T | Chr18 | 11,988,139 | T:G | 2,690 | 0.614228 | 0.954094 | 1.30E-36 |
|  |  | Chr18 | 12,004,584 | G:A | Chr18 | 11,934,773 | C:T | 69,811 | 0.066639 | 0.755765 | 2.42E-04 |
|  |  | Chr18 | 12,004,584 | G:A | Chr18 | 11,956,877 | C:A | 47,707 | 0.006884 | 1 | 0.36999 |
|  |  | Chr18 | 12,004,584 | G:A | Chr18 | 11,960,134 | G:T | 44,450 | 0.012398 | 0.715909 | 0.18091 |
|  |  | Chr18 | 12,004,584 | G:A | Chr18 | 11,966,880 | G:A | 37,704 | 0.052025 | 0.641975 | 0.00134 |
|  |  | Chr18 | 12,004,584 | G:A | Chr18 | 11,973,840 | C:T | 30,744 | 0.005242 | 1 | 0.60649 |
|  |  | Chr18 | 12,004,584 | G:A | Chr18 | 11,988,139 | T:G | 16,445 | 0.047938 | 0.63522 | 0.0019 |
|  |  | Chr18 | 12,004,584 | G:A | Chr18 | 11,990,829 | C:T | 13,755 | 0.025968 | 0.575368 | 0.03049 |
|  |  | Chr18 | 12,010,080 | G:A | Chr18 | 11,934,773 | C:T | 75,307 | 0.02577 | 0.402059 | 0.02141 |
|  |  | Chr18 | 12,010,080 | G:A | Chr18 | 11,956,877 | C:A | 53,203 | 0.011335 | 0.173418 | 0.14918 |
|  |  | Chr18 | 12,010,080 | G:A | Chr18 | 11,960,134 | G:T | 49,946 | 6.67E-04 | 0.063632 | 0.74336 |
|  |  | Chr18 | 12,010,080 | G:A | Chr18 | 11,966,880 | G:A | 43,200 | 0.032783 | 0.475232 | 0.00722 |
|  |  | Chr18 | 12,010,080 | G:A | Chr18 | 11,973,840 | C:T | 36,240 | 0.010314 | 0.191617 | 0.17738 |
|  |  | Chr18 | 12,010,080 | G:A | Chr18 | 11,988,139 | T:G | 21,941 | 0.03045 | 0.44335 | 0.00855 |
|  |  | Chr18 | 12,010,080 | G:A | Chr18 | 11,990,829 | C:T | 19,251 | 0.058628 | 0.494003 | 3.16E-04 |
|  |  | Chr18 | 12,010,080 | G:A | Chr18 | 12,004,584 | G:A | 5,496 | 0.019359 | 1 | 0.03966 |
|  |  | Chr18 | 12,012,682 | C:T | Chr18 | 11,934,773 | C:T | 77,909 | 0.038109 | 0.671621 | 0.00591 |
|  |  | Chr18 | 12,012,682 | C:T | Chr18 | 11,956,877 | C:A | 55,805 | 0.00492 | 1 | 0.60246 |
|  |  | Chr18 | 12,012,682 | C:T | Chr18 | 11,960,134 | G:T | 52,548 | 0.006552 | 0.611111 | 0.44165 |
|  |  | Chr18 | 12,012,682 | C:T | Chr18 | 11,966,880 | G:A | 45,802 | 0.042141 | 0.679167 | 0.00424 |
|  |  | Chr18 | 12,012,682 | C:T | Chr18 | 11,973,840 | C:T | 38,842 | 0.003915 | 1 | 1 |
|  |  | Chr18 | 12,012,682 | C:T | Chr18 | 11,988,139 | T:G | 24,543 | 0.039841 | 0.674452 | 0.00523 |
|  |  | Chr18 | 12,012,682 | C:T | Chr18 | 11,990,829 | C:T | 21,853 | 0.02175 | 0.618574 | 0.03646 |
|  |  | Chr18 | 12,012,682 | C:T | Chr18 | 12,004,584 | G:A | 8,098 | 0.739865 | 1 | 4.99E-14 |
|  |  | Chr18 | 12,012,682 | C:T | Chr18 | 12,010,080 | G:A | 2,602 | 0.014471 | 1 | 0.11612 |
|  |  | Chr18 | 12,015,165 | G:A | Chr18 | 11,934,773 | C:T | 80,392 | 0.065598 | 0.753247 | 2.97E-04 |
|  |  | Chr18 | 12,015,165 | G:A | Chr18 | 11,956,877 | C:A | 58,288 | 0.006908 | 1 | 0.37046 |
|  |  | Chr18 | 12,015,165 | G:A | Chr18 | 11,960,134 | G:T | 55,031 | 0.012531 | 0.714286 | 0.1802 |
|  |  | Chr18 | 12,015,165 | G:A | Chr18 | 11,966,880 | G:A | 48,285 | 0.049639 | 0.636218 | 0.0018 |
|  |  | Chr18 | 12,015,165 | G:A | Chr18 | 11,973,840 | C:T | 41,325 | 0.004605 | 1 | 0.60651 |
|  |  | Chr18 | 12,015,165 | G:A | Chr18 | 11,988,139 | T:G | 27,026 | 0.048075 | 0.633117 | 0.00209 |
|  |  | Chr18 | 12,015,165 | G:A | Chr18 | 11,990,829 | C:T | 24,336 | 0.025203 | 0.570076 | 0.03103 |
|  |  | Chr18 | 12,015,165 | G:A | Chr18 | 12,004,584 | G:A | 10,581 | 1 | 1 | 3.44E-20 |
|  |  | Chr18 | 12,015,165 | G:A | Chr18 | 12,010,080 | G:A | 5,085 | 0.019487 | 1 | 0.03994 |
|  |  | Chr18 | 12,015,165 | G:A | Chr18 | 12,012,682 | C:T | 2,483 | 0.739631 | 1 | 6.10E-14 |
|  |  | Chr18 | 12,034,002 | T:C | Chr18 | 11,934,773 | C:T | 99,229 | 0.004432 | 0.119733 | 0.31927 |
|  |  | Chr18 | 12,034,002 | T:C | Chr18 | 11,956,877 | C:A | 77,125 | 0.013123 | 0.258193 | 0.09601 |
|  |  | Chr18 | 12,034,002 | T:C | Chr18 | 11,960,134 | G:T | 73,868 | 0.001383 | 0.067862 | 0.65964 |
|  |  | Chr18 | 12,034,002 | T:C | Chr18 | 11,966,880 | G:A | 67,122 | 0.017519 | 0.246739 | 0.05698 |
|  |  | Chr18 | 12,034,002 | T:C | Chr18 | 11,973,840 | C:T | 60,162 | 0.001168 | 0.134409 | 0.64317 |
|  |  | Chr18 | 12,034,002 | T:C | Chr18 | 11,988,139 | T:G | 45,863 | 0.019685 | 0.251436 | 0.04304 |
|  |  | Chr18 | 12,034,002 | T:C | Chr18 | 11,990,829 | C:T | 43,173 | 0.025213 | 0.233524 | 0.02009 |
|  |  | Chr18 | 12,034,002 | T:C | Chr18 | 12,004,584 | G:A | 29,418 | 0.082787 | 1 | 1.01E-05 |
|  |  | Chr18 | 12,034,002 | T:C | Chr18 | 12,010,080 | G:A | 23,922 | 0.049405 | 0.317372 | 0.00106 |
|  |  | Chr18 | 12,034,002 | T:C | Chr18 | 12,012,682 | C:T | 21,320 | 0.063348 | 1 | 1.67E-04 |
|  |  | Chr18 | 12,034,002 | T:C | Chr18 | 12,015,165 | G:A | 18,837 | 0.086317 | 1 | 8.62E-06 |
|  |  | Chr18 | 12,039,412 | C:T | Chr18 | 11,956,877 | C:A | 82,535 | 0.002859 | 1 | 1 |
|  |  | Chr18 | 12,039,412 | C:T | Chr18 | 11,960,134 | G:T | 79,278 | 0.001345 | 0.36 | 1 |
|  |  | Chr18 | 12,039,412 | C:T | Chr18 | 11,966,880 | G:A | 72,532 | 0.049673 | 1 | 0.00259 |
|  |  | Chr18 | 12,039,412 | C:T | Chr18 | 11,973,840 | C:T | 65,572 | 0.002176 | 1 | 1 |
|  |  | Chr18 | 12,039,412 | C:T | Chr18 | 11,988,139 | T:G | 51,273 | 0.047607 | 1 | 0.00306 |
|  |  | Chr18 | 12,039,412 | C:T | Chr18 | 11,990,829 | C:T | 48,583 | 0.03139 | 1 | 0.01179 |
|  |  | Chr18 | 12,039,412 | C:T | Chr18 | 12,004,584 | G:A | 34,828 | 0.403646 | 1 | 1.58E-07 |
|  |  | Chr18 | 12,039,412 | C:T | Chr18 | 12,010,080 | G:A | 29,332 | 0.007793 | 1 | 0.33267 |
|  |  | Chr18 | 12,039,412 | C:T | Chr18 | 12,012,682 | C:T | 26,730 | 0.54559 | 1 | 2.56E-08 |
|  |  | Chr18 | 12,039,412 | C:T | Chr18 | 12,015,165 | G:A | 24,247 | 0.403349 | 1 | 1.76E-07 |
|  |  | Chr18 | 12,039,412 | C:T | Chr18 | 12,034,002 | T:C | 5,410 | 0.033755 | 1 | 0.00947 |
|  |  | Chr18 | 12,044,370 | C:A | Chr18 | 11,956,877 | C:A | 87,493 | 0.007492 | 1 | 0.37098 |
|  |  | Chr18 | 12,044,370 | C:A | Chr18 | 11,960,134 | G:T | 84,236 | 0.006087 | 0.479167 | 0.34642 |
|  |  | Chr18 | 12,044,370 | C:A | Chr18 | 11,966,880 | G:A | 77,490 | 0.041704 | 0.556617 | 0.00367 |
|  |  | Chr18 | 12,044,370 | C:A | Chr18 | 11,973,840 | C:T | 70,530 | 0.005706 | 1 | 0.61085 |
|  |  | Chr18 | 12,044,370 | C:A | Chr18 | 11,988,139 | T:G | 56,231 | 0.039064 | 0.550146 | 0.00473 |
|  |  | Chr18 | 12,044,370 | C:A | Chr18 | 11,990,829 | C:T | 53,541 | 0.018802 | 0.473504 | 0.0452 |
|  |  | Chr18 | 12,044,370 | C:A | Chr18 | 12,004,584 | G:A | 39,786 | 0.918881 | 1 | 3.42E-19 |
|  |  | Chr18 | 12,044,370 | C:A | Chr18 | 12,010,080 | G:A | 34,290 | 0.021071 | 1 | 0.02398 |
|  |  | Chr18 | 12,044,370 | C:A | Chr18 | 12,012,682 | C:T | 31,688 | 0.67989 | 1 | 1.56E-13 |
|  |  | Chr18 | 12,044,370 | C:A | Chr18 | 12,015,165 | G:A | 29,205 | 0.918784 | 1 | 4.47E-19 |
|  |  | Chr18 | 12,044,370 | C:A | Chr18 | 12,034,002 | T:C | 10,368 | 0.091743 | 1 | 3.16E-06 |
|  |  | Chr18 | 12,044,370 | C:A | Chr18 | 12,039,412 | C:T | 4,958 | 0.370879 | 1 | 2.56E-07 |
|  |  | Chr18 | 12,051,372 | C:T | Chr18 | 11,956,877 | C:A | 94,495 | 0.009261 | 1 | 0.22793 |
|  |  | Chr18 | 12,051,372 | C:T | Chr18 | 11,960,134 | G:T | 91,238 | 0.006031 | 0.411458 | 0.40342 |
|  |  | Chr18 | 12,051,372 | C:T | Chr18 | 11,966,880 | G:A | 84,492 | 0.050991 | 0.55054 | 0.00124 |
|  |  | Chr18 | 12,051,372 | C:T | Chr18 | 11,973,840 | C:T | 77,532 | 0.007054 | 1 | 0.37259 |
|  |  | Chr18 | 12,051,372 | C:T | Chr18 | 11,988,139 | T:G | 63,233 | 0.047751 | 0.544025 | 0.00169 |
|  |  | Chr18 | 12,051,372 | C:T | Chr18 | 11,990,829 | C:T | 60,543 | 0.048544 | 0.680147 | 0.00109 |
|  |  | Chr18 | 12,051,372 | C:T | Chr18 | 12,004,584 | G:A | 46,788 | 0.008154 | 0.105223 | 0.19486 |
|  |  | Chr18 | 12,051,372 | C:T | Chr18 | 12,010,080 | G:A | 41,292 | 0.037569 | 0.412352 | 0.00662 |
|  |  | Chr18 | 12,051,372 | C:T | Chr18 | 12,012,682 | C:T | 38,690 | 0.001116 | 0.045267 | 0.48052 |
|  |  | Chr18 | 12,051,372 | C:T | Chr18 | 12,015,165 | G:A | 36,207 | 0.007927 | 0.103774 | 0.20168 |
|  |  | Chr18 | 12,051,372 | C:T | Chr18 | 12,034,002 | T:C | 17,370 | 0.027093 | 0.526531 | 0.02097 |
|  |  | Chr18 | 12,051,372 | C:T | Chr18 | 12,039,412 | C:T | 11,960 | 0.005841 | 0.140187 | 0.30498 |
|  |  | Chr18 | 12,051,372 | C:T | Chr18 | 12,044,370 | C:A | 7,002 | 0.006703 | 0.091457 | 0.22123 |
|  |  | Chr18 | 12,061,236 | T:C | Chr18 | 11,966,880 | G:A | 94,356 | 0.02701 | 0.47493 | 0.01461 |
|  |  | Chr18 | 12,061,236 | T:C | Chr18 | 11,973,840 | C:T | 87,396 | 0.003983 | 0.107692 | 0.39217 |
|  |  | Chr18 | 12,061,236 | T:C | Chr18 | 11,988,139 | T:G | 73,097 | 0.023868 | 0.433243 | 0.02502 |
|  |  | Chr18 | 12,061,236 | T:C | Chr18 | 11,990,829 | C:T | 70,407 | 0.008833 | 0.215653 | 0.19205 |
|  |  | Chr18 | 12,061,236 | T:C | Chr18 | 12,004,584 | G:A | 56,652 | 0.014836 | 1 | 0.07459 |
|  |  | Chr18 | 12,061,236 | T:C | Chr18 | 12,010,080 | G:A | 51,156 | 3.57E-04 | 0.06069 | 0.85567 |
|  |  | Chr18 | 12,061,236 | T:C | Chr18 | 12,012,682 | C:T | 48,554 | 0.010806 | 1 | 0.21025 |
|  |  | Chr18 | 12,061,236 | T:C | Chr18 | 12,015,165 | G:A | 46,071 | 0.014426 | 1 | 0.1334 |
|  |  | Chr18 | 12,061,236 | T:C | Chr18 | 12,034,002 | T:C | 27,234 | 0.016641 | 0.201143 | 0.05256 |
|  |  | Chr18 | 12,061,236 | T:C | Chr18 | 12,039,412 | C:T | 21,824 | 0.006016 | 1 | 0.58718 |
|  |  | Chr18 | 12,061,236 | T:C | Chr18 | 12,044,370 | C:A | 16,866 | 0.015736 | 1 | 0.07575 |
|  |  | Chr18 | 12,061,236 | T:C | Chr18 | 12,051,372 | C:T | 9,864 | 0.019944 | 1 | 0.02664 |
|  |  | Chr18 | 12,094,075 | G:A | Chr18 | 12,004,584 | G:A | 89,491 | 0.006235 | 1 | 0.61843 |
|  |  | Chr18 | 12,094,075 | G:A | Chr18 | 12,010,080 | G:A | 83,995 | 8.61E-04 | 0.149175 | 0.80343 |
|  |  | Chr18 | 12,094,075 | G:A | Chr18 | 12,012,682 | C:T | 81,393 | 0.004657 | 1 | 0.60316 |
|  |  | Chr18 | 12,094,075 | G:A | Chr18 | 12,015,165 | G:A | 78,910 | 0.006233 | 1 | 0.61602 |
|  |  | Chr18 | 12,094,075 | G:A | Chr18 | 12,034,002 | T:C | 60,073 | 0.076282 | 1 | 2.64E-06 |
|  |  | Chr18 | 12,094,075 | G:A | Chr18 | 12,039,412 | C:T | 54,663 | 0.00235 | 1 | 1 |
|  |  | Chr18 | 12,094,075 | G:A | Chr18 | 12,044,370 | C:A | 49,705 | 0.006786 | 1 | 0.37153 |
|  |  | Chr18 | 12,094,075 | G:A | Chr18 | 12,051,372 | C:T | 42,703 | 0.008388 | 1 | 0.38365 |
|  |  | Chr18 | 12,094,075 | G:A | Chr18 | 12,061,236 | T:C | 32,839 | 0.005231 | 0.4125 | 0.42856 |
|  |  |  |  |  |  |  |  |  |  |  |  |
| ss715631131 | 46,643,373 | Chr18 | 46,640,732 | C:T | Chr18 | 46,612,247 | A:C | 28,485 | 0.019847 | 0.311839 | 0.0531 |
|  |  | Chr18 | 46,643,373 | G:A | Chr18 | 46,612,247 | A:C | 31,126 | 0.003774 | 1 | 1 |
|  |  | Chr18 | 46,643,373 | G:A | Chr18 | 46,640,732 | C:T | 2,641 | 0.251505 | 1 | 4.95E-11 |
|  |  | Chr18 | 46,669,339 | T:C | Chr18 | 46,612,247 | A:C | 57,092 | 0.041298 | 0.329467 | 0.0109 |
|  |  | Chr18 | 46,669,339 | T:C | Chr18 | 46,640,732 | C:T | 28,607 | 0.466916 | 0.912825 | 5.53E-20 |
|  |  | Chr18 | 46,669,339 | T:C | Chr18 | 46,643,373 | G:A | 25,966 | 0.432416 | 1 | 1.45E-14 |
|  |  | Chr18 | 46,684,006 | G:A | Chr18 | 46,612,247 | A:C | 71,759 | 6.90E-04 | 0.293333 | 1 |
|  |  | Chr18 | 46,684,006 | G:A | Chr18 | 46,640,732 | C:T | 43,274 | 0.024983 | 0.816522 | 0.01954 |
|  |  | Chr18 | 46,684,006 | G:A | Chr18 | 46,643,373 | G:A | 40,633 | 0.009701 | 1 | 0.22591 |
|  |  | Chr18 | 46,684,006 | G:A | Chr18 | 46,669,339 | T:C | 14,667 | 0.022798 | 1 | 0.0314 |
|  |  |  |  |  |  |  |  |  |  |  |  |
| ss715631193 | 47,125,551 | Chr18 | 47,101,079 | G:A | Chr18 | 47,071,111 | C:T | 29,968 | 0.030379 | 1 | 0.01256 |
|  |  | Chr18 | 47,125,551 | C:T | Chr18 | 47,071,111 | C:T | 54,440 | 0.326633 | 1 | 7.46E-04 |
|  |  | Chr18 | 47,125,551 | C:T | Chr18 | 47,101,079 | G:A | 24,472 | 0.002497 | 0.372549 | 0.60079 |
|  |  | Chr18 | 47,136,557 | G:T | Chr18 | 47,071,111 | C:T | 65,446 | 0.287936 | 0.58914 | 1.02E-04 |
|  |  | Chr18 | 47,136,557 | G:T | Chr18 | 47,101,079 | G:A | 35,478 | 0.033936 | 1 | 0.00641 |
|  |  | Chr18 | 47,136,557 | G:T | Chr18 | 47,125,551 | C:T | 11,006 | 0.180213 | 0.654941 | 0.00293 |
|  |  |  |  |  |  |  |  |  |  |  |  |
| ss715631383 | 48,495,551 | Chr18 | 48,468,241 | A:G | Chr18 | 48,457,688 | T:C | 10,553 | 0.021815 | 1 | 0.01776 |
|  |  | Chr18 | 48,470,291 | A:G | Chr18 | 48,457,688 | T:C | 12,603 | 0.128194 | 0.43358 | 5.46E-07 |
|  |  | Chr18 | 48,470,291 | A:G | Chr18 | 48,468,241 | A:G | 2,050 | 0.363208 | 1 | 6.35E-17 |
|  |  | Chr18 | 48,473,402 | G:N | Chr18 | 48,457,688 | T:C | 15,714 | NaN | NaN | NaN |
|  |  | Chr18 | 48,473,402 | G:N | Chr18 | 48,468,241 | A:G | 5,161 | NaN | NaN | NaN |
|  |  | Chr18 | 48,473,402 | G:N | Chr18 | 48,470,291 | A:G | 3,111 | NaN | NaN | NaN |
|  |  | Chr18 | 48,491,469 | C:T | Chr18 | 48,457,688 | T:C | 33,781 | 0.00717 | 0.111428 | 0.23715 |
|  |  | Chr18 | 48,491,469 | C:T | Chr18 | 48,468,241 | A:G | 23,228 | 0.013333 | 1 | 0.14134 |
|  |  | Chr18 | 48,491,469 | C:T | Chr18 | 48,470,291 | A:G | 21,178 | 0.03576 | 1 | 0.00172 |
|  |  | Chr18 | 48,491,469 | C:T | Chr18 | 48,473,402 | G:N | 18,067 | NaN | NaN | NaN |
|  |  | Chr18 | 48,495,551 | C:A | Chr18 | 48,457,688 | T:C | 37,863 | 0.013366 | 1 | 0.13552 |
|  |  | Chr18 | 48,495,551 | C:A | Chr18 | 48,468,241 | A:G | 27,310 | 0.474544 | 0.915057 | 2.01E-13 |
|  |  | Chr18 | 48,495,551 | C:A | Chr18 | 48,470,291 | A:G | 25,260 | 0.217324 | 1 | 2.11E-10 |
|  |  | Chr18 | 48,495,551 | C:A | Chr18 | 48,473,402 | G:N | 22,149 | NaN | NaN | NaN |
|  |  | Chr18 | 48,495,551 | C:A | Chr18 | 48,491,469 | C:T | 4,082 | 0.008079 | 1 | 0.37294 |
|  |  | Chr18 | 48,506,101 | T:C | Chr18 | 48,457,688 | T:C | 48,413 | 0.003513 | 0.08327 | 0.36486 |
|  |  | Chr18 | 48,506,101 | T:C | Chr18 | 48,468,241 | A:G | 37,860 | 0.011047 | 1 | 0.23636 |
|  |  | Chr18 | 48,506,101 | T:C | Chr18 | 48,470,291 | A:G | 35,810 | 0.031051 | 1 | 0.00506 |
|  |  | Chr18 | 48,506,101 | T:C | Chr18 | 48,473,402 | G:N | 32,699 | NaN | NaN | NaN |
|  |  | Chr18 | 48,506,101 | T:C | Chr18 | 48,491,469 | C:T | 14,632 | 1 | 1 | 1.08E-29 |
|  |  | Chr18 | 48,506,101 | T:C | Chr18 | 48,495,551 | C:A | 10,550 | 0.006765 | 1 | 0.37335 |
|  |  | Chr18 | 48,509,246 | C:T | Chr18 | 48,457,688 | T:C | 51,558 | 0.007726 | 0.162531 | 0.24788 |
|  |  | Chr18 | 48,509,246 | C:T | Chr18 | 48,468,241 | A:G | 41,005 | 0.006396 | 1 | 0.62004 |
|  |  | Chr18 | 48,509,246 | C:T | Chr18 | 48,470,291 | A:G | 38,955 | 0.017939 | 1 | 0.04286 |
|  |  | Chr18 | 48,509,246 | C:T | Chr18 | 48,473,402 | G:N | 35,844 | NaN | NaN | NaN |
|  |  | Chr18 | 48,509,246 | C:T | Chr18 | 48,491,469 | C:T | 17,777 | 0.627805 | 1 | 2.77E-16 |
|  |  | Chr18 | 48,509,246 | C:T | Chr18 | 48,495,551 | C:A | 13,695 | 0.003919 | 1 | 1 |
|  |  | Chr18 | 48,509,246 | C:T | Chr18 | 48,506,101 | T:C | 3,145 | 0.689076 | 1 | 2.80E-16 |
|  |  | Chr18 | 48,511,290 | C:N | Chr18 | 48,457,688 | T:C | 53,602 | NaN | NaN | NaN |
|  |  | Chr18 | 48,511,290 | C:N | Chr18 | 48,468,241 | A:G | 43,049 | NaN | NaN | NaN |
|  |  | Chr18 | 48,511,290 | C:N | Chr18 | 48,470,291 | A:G | 40,999 | NaN | NaN | NaN |
|  |  | Chr18 | 48,511,290 | C:N | Chr18 | 48,473,402 | G:N | 37,888 | NaN | NaN | NaN |
|  |  | Chr18 | 48,511,290 | C:N | Chr18 | 48,491,469 | C:T | 19,821 | NaN | NaN | NaN |
|  |  | Chr18 | 48,511,290 | C:N | Chr18 | 48,495,551 | C:A | 15,739 | NaN | NaN | NaN |
|  |  | Chr18 | 48,511,290 | C:N | Chr18 | 48,506,101 | T:C | 5,189 | NaN | NaN | NaN |
|  |  | Chr18 | 48,511,290 | C:N | Chr18 | 48,509,246 | C:T | 2,044 | NaN | NaN | NaN |
|  |  |  |  |  |  |  |  |  |  |  |  |
| ss715631522 | 49,659,228 | Chr18 | 49,614,370 | G:A | Chr18 | 49,582,012 | C:T | 32,358 | 0.013594 | 1 | 0.11094 |
|  |  | Chr18 | 49,628,626 | A:G | Chr18 | 49,582,012 | C:T | 46,614 | 0.092907 | 0.702564 | 2.50E-04 |
|  |  | Chr18 | 49,628,626 | A:G | Chr18 | 49,614,370 | G:A | 14,256 | 0.019693 | 0.513085 | 0.04362 |
|  |  | Chr18 | 49,634,768 | G:T | Chr18 | 49,582,012 | C:T | 52,756 | 0.011217 | 1 | 0.2031 |
|  |  | Chr18 | 49,634,768 | G:T | Chr18 | 49,614,370 | G:A | 20,398 | 0.007635 | 0.091925 | 0.19937 |
|  |  | Chr18 | 49,634,768 | G:T | Chr18 | 49,628,626 | A:G | 6,142 | 0.03256 | 0.752 | 0.00745 |
|  |  | Chr18 | 49,649,522 | C:T | Chr18 | 49,582,012 | C:T | 67,510 | 0.015414 | 0.44724 | 0.11504 |
|  |  | Chr18 | 49,649,522 | C:T | Chr18 | 49,614,370 | G:A | 35,152 | 0.16703 | 1 | 3.30E-12 |
|  |  | Chr18 | 49,649,522 | C:T | Chr18 | 49,628,626 | A:G | 20,896 | 0.00818 | 0.142157 | 0.1786 |
|  |  | Chr18 | 49,649,522 | C:T | Chr18 | 49,634,768 | G:T | 14,754 | 0.149343 | 1 | 1.17E-10 |
|  |  | Chr18 | 49,659,228 | A:G | Chr18 | 49,582,012 | C:T | 77,216 | 0.06822 | 0.562814 | 0.00179 |
|  |  | Chr18 | 49,659,228 | A:G | Chr18 | 49,614,370 | G:A | 44,858 | 0.005564 | 0.302611 | 0.29056 |
|  |  | Chr18 | 49,659,228 | A:G | Chr18 | 49,628,626 | A:G | 30,602 | 0.113487 | 0.366904 | 7.03E-06 |
|  |  | Chr18 | 49,659,228 | A:G | Chr18 | 49,634,768 | G:T | 24,460 | 0.027504 | 0.72875 | 0.01224 |
|  |  | Chr18 | 49,659,228 | A:G | Chr18 | 49,649,522 | C:T | 9,706 | 0.136995 | 0.629934 | 1.11E-07 |
|  |  | Chr18 | 49,666,529 | C:T | Chr18 | 49,582,012 | C:T | 84,517 | 0.074489 | 0.567164 | 0.00131 |
|  |  | Chr18 | 49,666,529 | C:T | Chr18 | 49,614,370 | G:A | 52,159 | 0.01275 | 0.486716 | 0.11618 |
|  |  | Chr18 | 49,666,529 | C:T | Chr18 | 49,628,626 | A:G | 37,903 | 0.108966 | 0.367185 | 1.26E-05 |
|  |  | Chr18 | 49,666,529 | C:T | Chr18 | 49,634,768 | G:T | 31,761 | 0.015674 | 0.568 | 0.0998 |
|  |  | Chr18 | 49,666,529 | C:T | Chr18 | 49,649,522 | C:T | 17,007 | 0.140548 | 0.65614 | 8.77E-08 |
|  |  | Chr18 | 49,666,529 | C:T | Chr18 | 49,659,228 | A:G | 7,301 | 0.962879 | 1 | 9.61E-37 |
|  |  | Chr18 | 49,669,867 | A:C | Chr18 | 49,582,012 | C:T | 87,855 | 0.002273 | 1 | 1 |
|  |  | Chr18 | 49,669,867 | A:C | Chr18 | 49,614,370 | G:A | 55,497 | 0.024434 | 1 | 0.01309 |
|  |  | Chr18 | 49,669,867 | A:C | Chr18 | 49,628,626 | A:G | 41,241 | 0.003777 | 0.554054 | 0.70463 |
|  |  | Chr18 | 49,669,867 | A:C | Chr18 | 49,634,768 | G:T | 35,099 | 0.076894 | 0.600733 | 3.17E-04 |
|  |  | Chr18 | 49,669,867 | A:C | Chr18 | 49,649,522 | C:T | 20,345 | 0.019379 | 0.779843 | 0.03997 |
|  |  | Chr18 | 49,669,867 | A:C | Chr18 | 49,659,228 | A:G | 10,639 | 0.010857 | 1 | 0.22987 |
|  |  | Chr18 | 49,669,867 | A:C | Chr18 | 49,666,529 | C:T | 3,338 | 0.010097 | 1 | 0.22569 |
|  |  | Chr18 | 49,673,204 | G:T | Chr18 | 49,582,012 | C:T | 91,192 | 0.064835 | 0.642371 | 0.00398 |
|  |  | Chr18 | 49,673,204 | G:T | Chr18 | 49,614,370 | G:A | 58,834 | 0.002282 | 0.18797 | 0.64712 |
|  |  | Chr18 | 49,673,204 | G:T | Chr18 | 49,628,626 | A:G | 44,578 | 0.188762 | 0.48701 | 1.95E-07 |
|  |  | Chr18 | 49,673,204 | G:T | Chr18 | 49,634,768 | G:T | 38,436 | 0.049193 | 0.769841 | 0.00213 |
|  |  | Chr18 | 49,673,204 | G:T | Chr18 | 49,649,522 | C:T | 23,682 | 0.091021 | 0.507003 | 8.59E-05 |
|  |  | Chr18 | 49,673,204 | G:T | Chr18 | 49,659,228 | A:G | 13,976 | 0.930116 | 1 | 3.63E-34 |
|  |  | Chr18 | 49,673,204 | G:T | Chr18 | 49,666,529 | C:T | 6,675 | 0.858979 | 1 | 2.92E-30 |
|  |  | Chr18 | 49,673,204 | G:T | Chr18 | 49,669,867 | A:C | 3,337 | 0.020994 | 1 | 0.07646 |
|  |  | Chr18 | 49,680,666 | C:T | Chr18 | 49,582,012 | C:T | 98,654 | 0.055805 | 0.552665 | 0.00335 |
|  |  | Chr18 | 49,680,666 | C:T | Chr18 | 49,614,370 | G:A | 66,296 | 0.00245 | 0.069429 | 0.54611 |
|  |  | Chr18 | 49,680,666 | C:T | Chr18 | 49,628,626 | A:G | 52,040 | 0.127374 | 0.356895 | 1.48E-06 |
|  |  | Chr18 | 49,680,666 | C:T | Chr18 | 49,634,768 | G:T | 45,898 | 0.036777 | 0.769474 | 0.00279 |
|  |  | Chr18 | 49,680,666 | C:T | Chr18 | 49,649,522 | C:T | 31,144 | 0.002022 | 0.069036 | 0.56862 |
|  |  | Chr18 | 49,680,666 | C:T | Chr18 | 49,659,228 | A:G | 21,438 | 0.082129 | 0.311465 | 9.30E-05 |
|  |  | Chr18 | 49,680,666 | C:T | Chr18 | 49,666,529 | C:T | 14,137 | 0.077701 | 0.309181 | 1.55E-04 |
|  |  | Chr18 | 49,680,666 | C:T | Chr18 | 49,669,867 | A:C | 10,799 | 0.012458 | 1 | 0.13423 |
|  |  | Chr18 | 49,680,666 | C:T | Chr18 | 49,673,204 | G:T | 7,462 | 0.116589 | 0.422292 | 3.16E-05 |
|  |  | Chr18 | 49,702,732 | C:T | Chr18 | 49,614,370 | G:A | 88,362 | 6.97E-05 | 0.021488 | 1 |
|  |  | Chr18 | 49,702,732 | C:T | Chr18 | 49,628,626 | A:G | 74,106 | 9.48E-05 | 0.019062 | 1 |
|  |  | Chr18 | 49,702,732 | C:T | Chr18 | 49,634,768 | G:T | 67,964 | 0.06761 | 0.615854 | 0.00114 |
|  |  | Chr18 | 49,702,732 | C:T | Chr18 | 49,649,522 | C:T | 53,210 | 0.026206 | 1 | 0.01757 |
|  |  | Chr18 | 49,702,732 | C:T | Chr18 | 49,659,228 | A:G | 43,504 | 0.008693 | 1 | 0.37215 |
|  |  | Chr18 | 49,702,732 | C:T | Chr18 | 49,666,529 | C:T | 36,203 | 0.008065 | 1 | 0.36761 |
|  |  | Chr18 | 49,702,732 | C:T | Chr18 | 49,669,867 | A:C | 32,865 | 7.11E-04 | 0.030303 | 0.51657 |
|  |  | Chr18 | 49,702,732 | C:T | Chr18 | 49,673,204 | G:T | 29,528 | 0.016484 | 1 | 0.12395 |
|  |  | Chr18 | 49,702,732 | C:T | Chr18 | 49,680,666 | C:T | 22,066 | 0.002182 | 0.45933 | 0.69556 |
|  |  |  |  |  |  |  |  |  |  |  |  |
| ss715634285 | 35,586,767 | Chr19 | 35,546,420 | C:T | Chr19 | 35,535,527 | G:A | 10,893 | 0.038556 | 0.780651 | 0.00163 |
|  |  | Chr19 | 35,546,970 | T:C | Chr19 | 35,535,527 | G:A | 11,443 | 0.021539 | 0.62965 | 0.02863 |
|  |  | Chr19 | 35,546,970 | T:C | Chr19 | 35,546,420 | C:T | 550 | 0.105731 | 1 | 1.42E-08 |
|  |  | Chr19 | 35,555,035 | T:C | Chr19 | 35,535,527 | G:A | 19,508 | 0.010358 | 0.421922 | 0.14166 |
|  |  | Chr19 | 35,555,035 | T:C | Chr19 | 35,546,420 | C:T | 8,615 | 0.105861 | 1 | 1.50E-08 |
|  |  | Chr19 | 35,555,035 | T:C | Chr19 | 35,546,970 | T:C | 8,065 | 0.835225 | 0.925226 | 1.07E-41 |
|  |  | Chr19 | 35,586,767 | G:T | Chr19 | 35,535,527 | G:A | 51,240 | 1.63E-05 | 0.006076 | 1 |
|  |  | Chr19 | 35,586,767 | G:T | Chr19 | 35,546,420 | C:T | 40,347 | 2.01E-04 | 0.02859 | 0.78424 |
|  |  | Chr19 | 35,586,767 | G:T | Chr19 | 35,546,970 | T:C | 39,797 | 4.08E-05 | 0.039832 | 1 |
|  |  | Chr19 | 35,586,767 | G:T | Chr19 | 35,555,035 | T:C | 31,732 | 0.002124 | 0.287037 | 0.57673 |
|  |  | Chr19 | 35,589,820 | G:T | Chr19 | 35,535,527 | G:A | 54,293 | 0.020095 | 0.306667 | 0.04225 |
|  |  | Chr19 | 35,589,820 | G:T | Chr19 | 35,546,420 | C:T | 43,400 | 0.113273 | 0.600228 | 3.38E-07 |
|  |  | Chr19 | 35,589,820 | G:T | Chr19 | 35,546,970 | T:C | 42,850 | 0.001654 | 0.079739 | 0.63246 |
|  |  | Chr19 | 35,589,820 | G:T | Chr19 | 35,555,035 | T:C | 34,785 | 5.97E-04 | 0.047985 | 0.75309 |
|  |  | Chr19 | 35,589,820 | G:T | Chr19 | 35,586,767 | G:T | 3,053 | 0.073433 | 1 | 1.24E-05 |
|  |  | Chr19 | 35,611,769 | C:T | Chr19 | 35,535,527 | G:A | 76,242 | 0.013766 | 0.224432 | 0.11263 |
|  |  | Chr19 | 35,611,769 | C:T | Chr19 | 35,546,420 | C:T | 65,349 | 0.011873 | 1 | 0.21649 |
|  |  | Chr19 | 35,611,769 | C:T | Chr19 | 35,546,970 | T:C | 64,799 | 0.064638 | 0.69697 | 0.00158 |
|  |  | Chr19 | 35,611,769 | C:T | Chr19 | 35,555,035 | T:C | 56,734 | 0.090688 | 0.751263 | 1.48E-04 |
|  |  | Chr19 | 35,611,769 | C:T | Chr19 | 35,586,767 | G:T | 25,002 | 0.00608 | 1 | 0.60342 |
|  |  | Chr19 | 35,611,769 | C:T | Chr19 | 35,589,820 | G:T | 21,949 | 0.066044 | 1 | 2.86E-04 |
|  |  | Chr19 | 35,627,532 | G:A | Chr19 | 35,535,527 | G:A | 92,005 | 0.081789 | 0.326019 | 1.29E-04 |
|  |  | Chr19 | 35,627,532 | G:A | Chr19 | 35,546,420 | C:T | 81,112 | 0.001686 | 0.046563 | 0.54263 |
|  |  | Chr19 | 35,627,532 | G:A | Chr19 | 35,546,970 | T:C | 80,562 | 0.015403 | 0.433962 | 0.07693 |
|  |  | Chr19 | 35,627,532 | G:A | Chr19 | 35,555,035 | T:C | 72,497 | 0.023235 | 0.53273 | 0.02937 |
|  |  | Chr19 | 35,627,532 | G:A | Chr19 | 35,586,767 | G:T | 40,765 | 0.011067 | 0.171484 | 0.12883 |
|  |  | Chr19 | 35,627,532 | G:A | Chr19 | 35,589,820 | G:T | 37,712 | 0.021895 | 0.299668 | 0.04747 |
|  |  | Chr19 | 35,627,532 | G:A | Chr19 | 35,611,769 | C:T | 15,763 | 0.014588 | 1 | 0.21806 |
|  |  |  |  |  |  |  |  |  |  |  |  |
| ss715634622 | 37,724,122 | Chr19 | 37,714,295 | T:C | Chr19 | 37,674,704 | G:A | 39,591 | 0.031887 | 0.524103 | 0.02989 |
|  |  | Chr19 | 37,724,122 | A:G | Chr19 | 37,674,704 | G:A | 49,418 | 0.039526 | 0.230961 | 0.00586 |
|  |  | Chr19 | 37,724,122 | A:G | Chr19 | 37,714,295 | T:C | 9,827 | 0.088346 | 1 | 3.05E-04 |
|  |  | Chr19 | 37,740,969 | G:A | Chr19 | 37,674,704 | G:A | 66,265 | 0.057737 | 0.553846 | 0.00313 |
|  |  | Chr19 | 37,740,969 | G:A | Chr19 | 37,714,295 | T:C | 26,674 | 0.616776 | 1 | 1.02E-08 |
|  |  | Chr19 | 37,740,969 | G:A | Chr19 | 37,724,122 | A:G | 16,847 | 0.067325 | 0.685393 | 0.00114 |
|  |  | Chr19 | 37,742,188 | G:A | Chr19 | 37,674,704 | G:A | 67,484 | 0.063115 | 1 | 2.32E-05 |
|  |  | Chr19 | 37,742,188 | G:A | Chr19 | 37,714,295 | T:C | 27,893 | 0.008203 | 1 | 0.3309 |
|  |  | Chr19 | 37,742,188 | G:A | Chr19 | 37,724,122 | A:G | 18,066 | 0.034513 | 0.629268 | 0.00576 |
|  |  | Chr19 | 37,742,188 | G:A | Chr19 | 37,740,969 | G:A | 1,219 | 0.013314 | 1 | 0.11631 |
|  |  | Chr19 | 37,749,392 | C:T | Chr19 | 37,674,704 | G:A | 74,688 | 0.039479 | 0.490525 | 0.01339 |
|  |  | Chr19 | 37,749,392 | C:T | Chr19 | 37,714,295 | T:C | 35,097 | 0.708047 | 1 | 3.75E-09 |
|  |  | Chr19 | 37,749,392 | C:T | Chr19 | 37,724,122 | A:G | 25,270 | 0.084105 | 0.820431 | 3.28E-04 |
|  |  | Chr19 | 37,749,392 | C:T | Chr19 | 37,740,969 | G:A | 8,423 | 0.871145 | 1 | 1.15E-12 |
|  |  | Chr19 | 37,749,392 | C:T | Chr19 | 37,742,188 | G:A | 7,204 | 0.011468 | 1 | 0.19473 |
|  |  | Chr19 | 37,769,580 | G:A | Chr19 | 37,674,704 | G:A | 94,876 | 8.98E-05 | 0.019274 | 1 |
|  |  | Chr19 | 37,769,580 | G:A | Chr19 | 37,714,295 | T:C | 55,285 | 0.022944 | 1 | 0.06887 |
|  |  | Chr19 | 37,769,580 | G:A | Chr19 | 37,724,122 | A:G | 45,458 | 0.056302 | 0.483683 | 0.00208 |
|  |  | Chr19 | 37,769,580 | G:A | Chr19 | 37,740,969 | G:A | 28,611 | 0.036775 | 1 | 0.01025 |
|  |  | Chr19 | 37,769,580 | G:A | Chr19 | 37,742,188 | G:A | 27,392 | 0.011835 | 0.152632 | 0.20232 |
|  |  | Chr19 | 37,769,580 | G:A | Chr19 | 37,749,392 | C:T | 20,188 | 0.031987 | 1 | 0.01933 |
|  |  | Chr19 | 37,801,955 | T:C | Chr19 | 37,714,295 | T:C | 87,660 | 0.005191 | 0.245055 | 0.27266 |
|  |  | Chr19 | 37,801,955 | T:C | Chr19 | 37,724,122 | A:G | 77,833 | 1.13E-04 | 0.010918 | 0.84107 |
|  |  | Chr19 | 37,801,955 | T:C | Chr19 | 37,740,969 | G:A | 60,986 | 4.45E-04 | 0.056319 | 0.66889 |
|  |  | Chr19 | 37,801,955 | T:C | Chr19 | 37,742,188 | G:A | 59,767 | 0.038504 | 0.223611 | 0.00662 |
|  |  | Chr19 | 37,801,955 | T:C | Chr19 | 37,749,392 | C:T | 52,563 | 0.001278 | 0.102264 | 0.63366 |
|  |  | Chr19 | 37,801,955 | T:C | Chr19 | 37,769,580 | G:A | 32,375 | 0.011216 | 0.223744 | 0.19893 |
