# Supplementary material for: Genome-Wide Association Study of Resistance to Soybean Cyst Nematode (Heterodera glycines) HG Type 2.5.7 in Wild Soybean (Glycine soja)
Source: Front Plant Sci. 2016 Aug 17;7:1214. doi: 10.3389/fpls.2016.01214 (PMC4987380; doi:10.3389/fpls.2016.01214)
Supplement: Supplementary file 2 [file Image1.PDF]

Supplementary Material

Genome-Wide Association Analysis of Resistance to Soybean Cyst Nematode (Heterodera glycines) HG Type 2.5.7 in Wild Soybean (Glycine soja)

Hengyou Zhang[1], Chunying Li[2], Eric L Davis[2], Jinshe Wang[3], Joshua D. Griffin[4], Janice Kofsky[1], and Bao-Hua Song[1]*

*** Correspondence:** Bao-Hua Song, bsong5@uncc.edu

Supplementary Figures

Figure S1 Distribution of FI among 235 *G. soja* accessions.

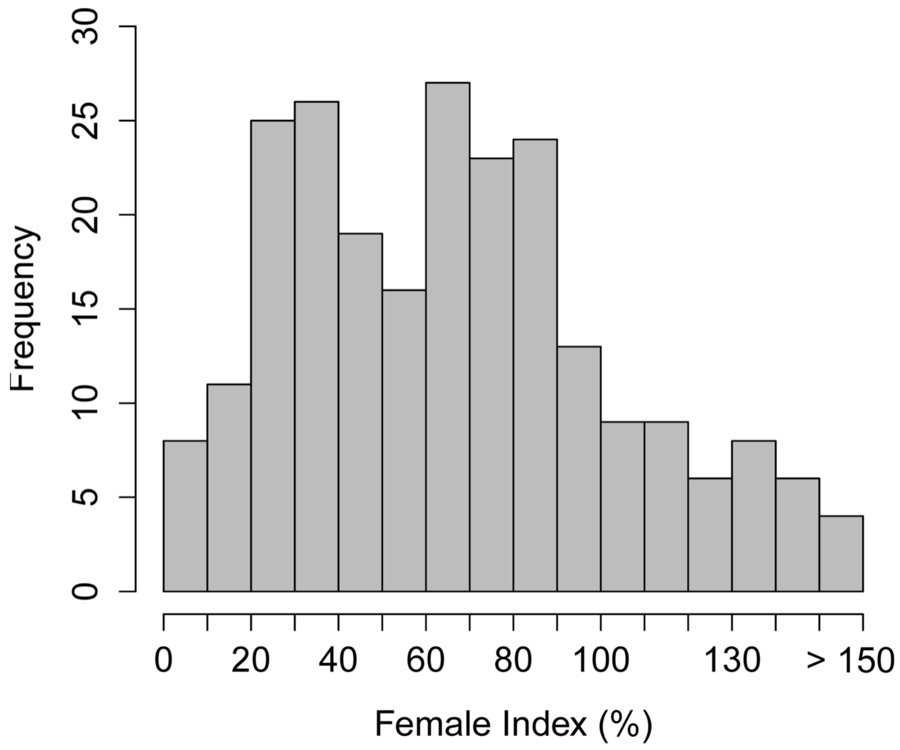

Figure S2 The distribution pattern of SoySNP 50 K SNP markers on 20 soybean chromosomes. The marker density is indicated by a colour index.

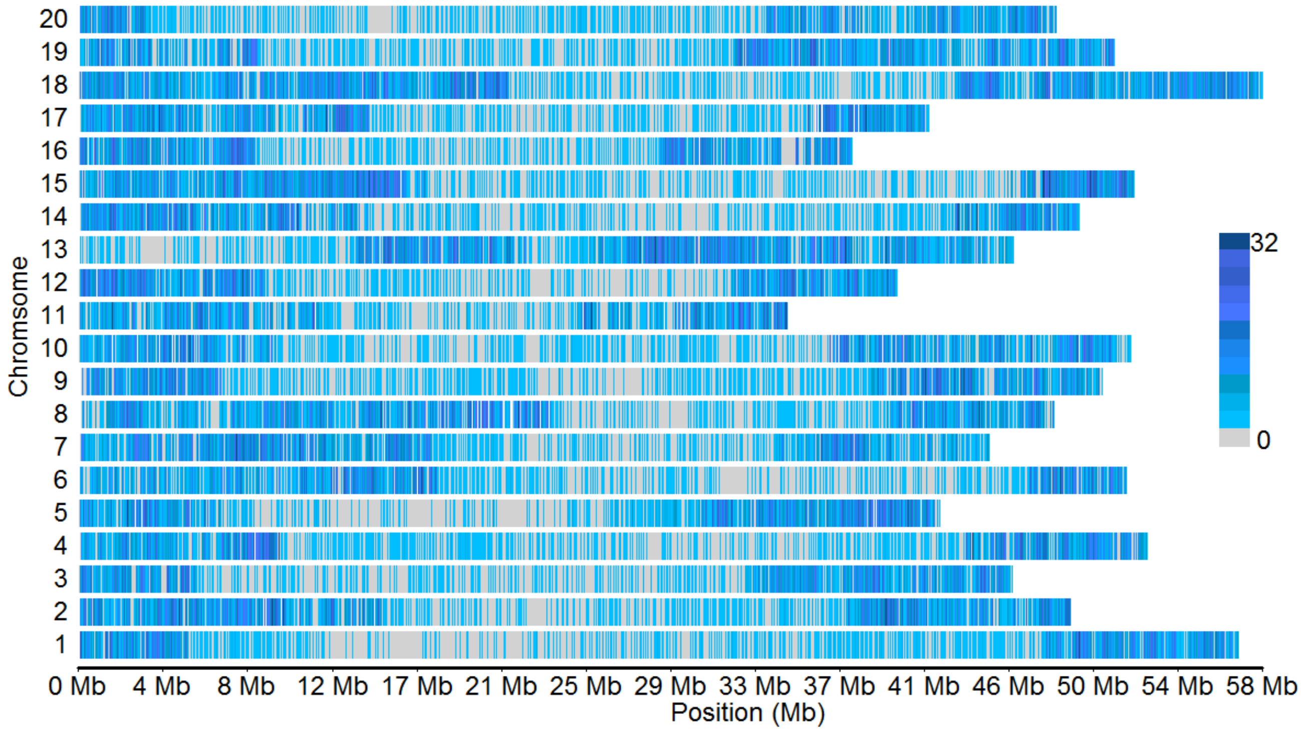

Figure S3 LD decay pattern of SNPs in each of 20 soybean chromosomes. Black solid dot on each LD line represents the average r^2 value that drops to half of the maximum LD value. The corresponding chromosome distance for each LD decay rate was provided aside.

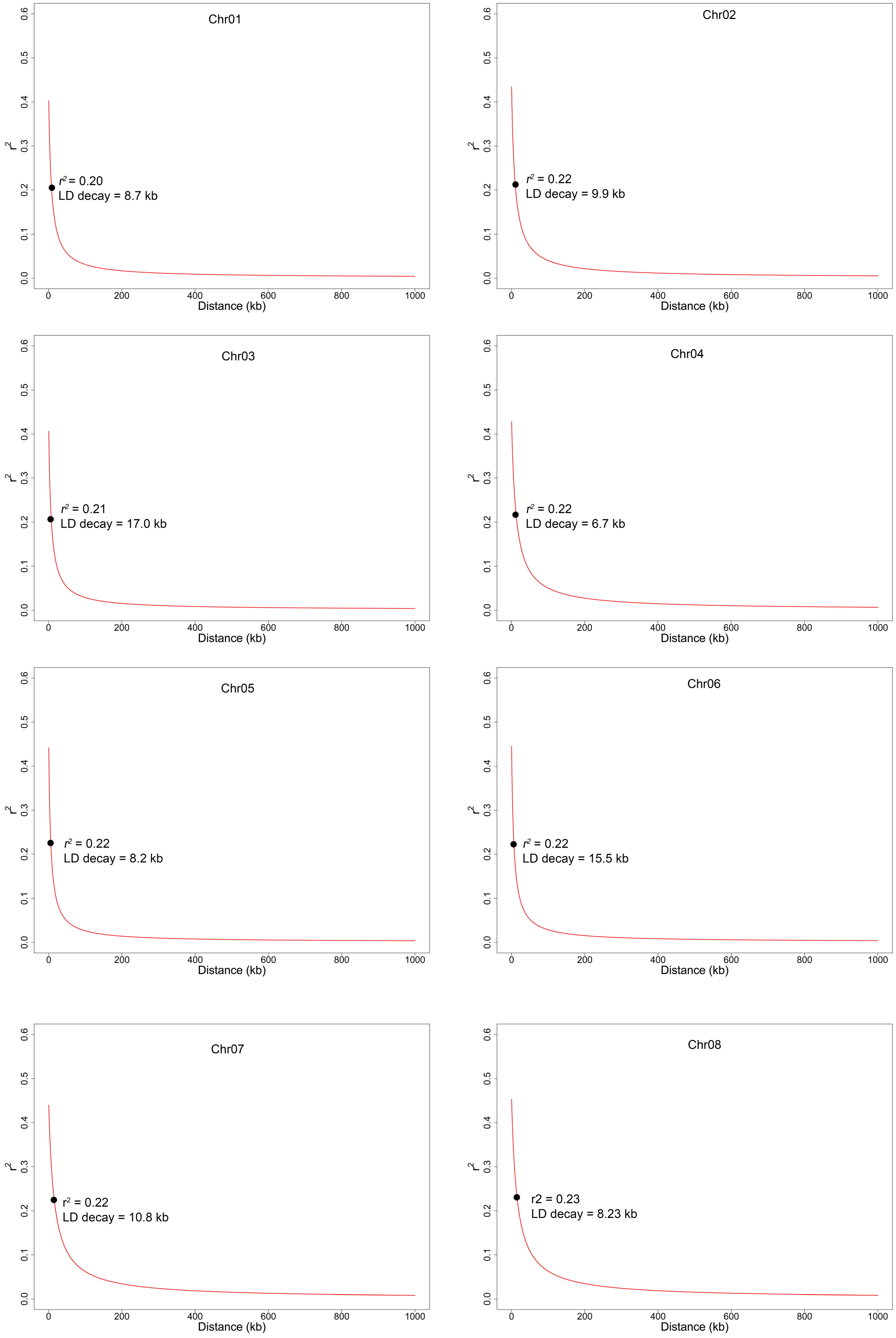
Chr01
r² = 0.20
LD decay = 8.7 kb
Chr02
r² = 0.22
LD decay = 9.9 kb
Chr03
r² = 0.21
LD decay = 17.0 kb
Chr04
r² = 0.22
LD decay = 6.7 kb
Chr05
r² = 0.22
LD decay = 8.2 kb
Chr06
r² = 0.22
LD decay = 15.5 kb
Chr07
r² = 0.22
LD decay = 10.8 kb
Chr08
r2 = 0.23
LD decay = 8.23 kb
r²
Distance (kb)
0.0 0.1 0.2 0.3 0.4 0.5 0.6
0 200 400 600 800 1000

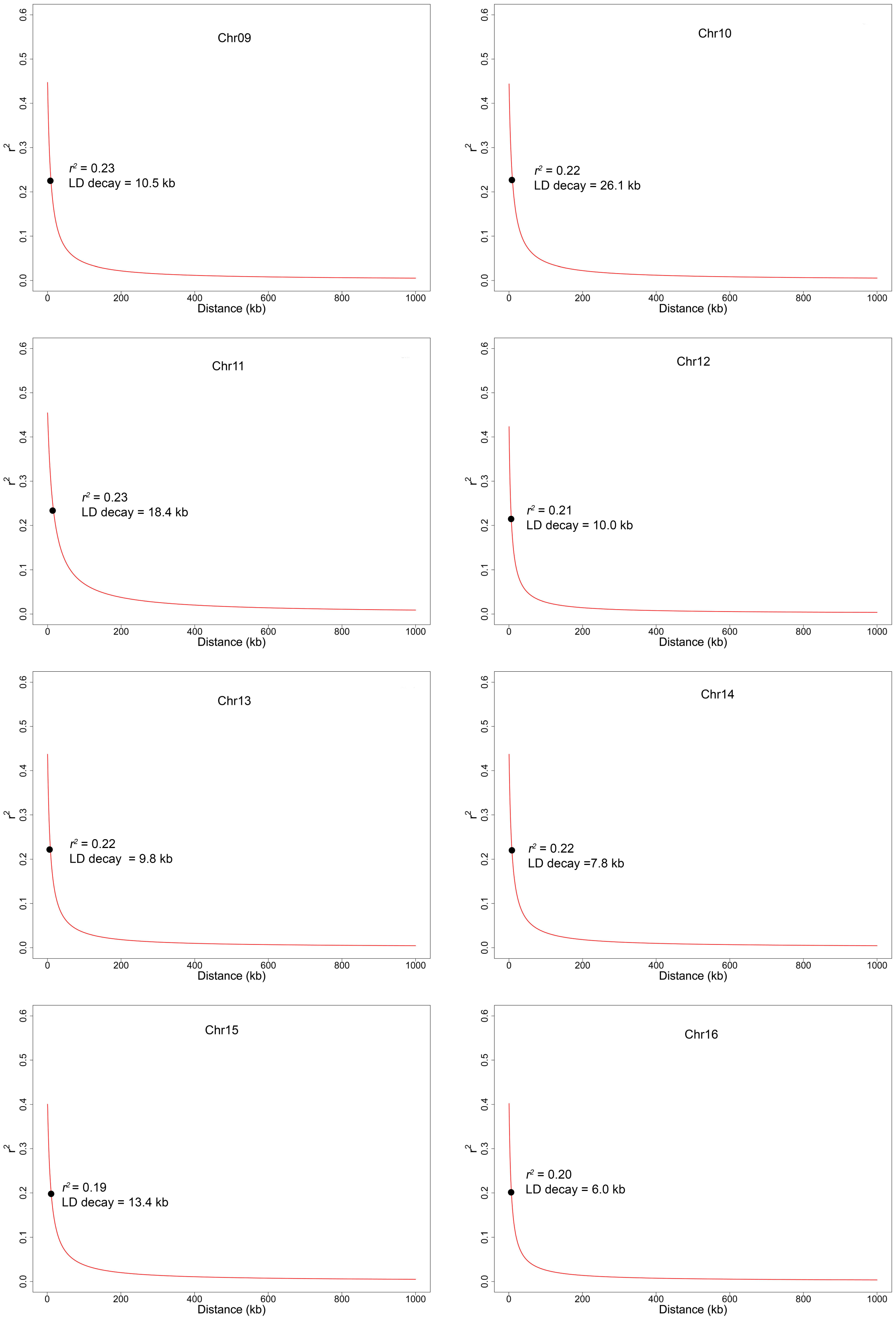
Chr09
r² = 0.23
LD decay = 10.5 kb
Chr10
r² = 0.22
LD decay = 26.1 kb
Chr11
r² = 0.23
LD decay = 18.4 kb
Chr12
r² = 0.21
LD decay = 10.0 kb
Chr13
r² = 0.22
LD decay = 9.8 kb
Chr14
r² = 0.22
LD decay =7.8 kb
Chr15
r² = 0.19
LD decay = 13.4 kb
Chr16
r² = 0.20
LD decay = 6.0 kb
r²
Distance (kb)
0.0
0.1
0.2
0.3
0.4
0.5
0.6
0
200
400
600
800
1000

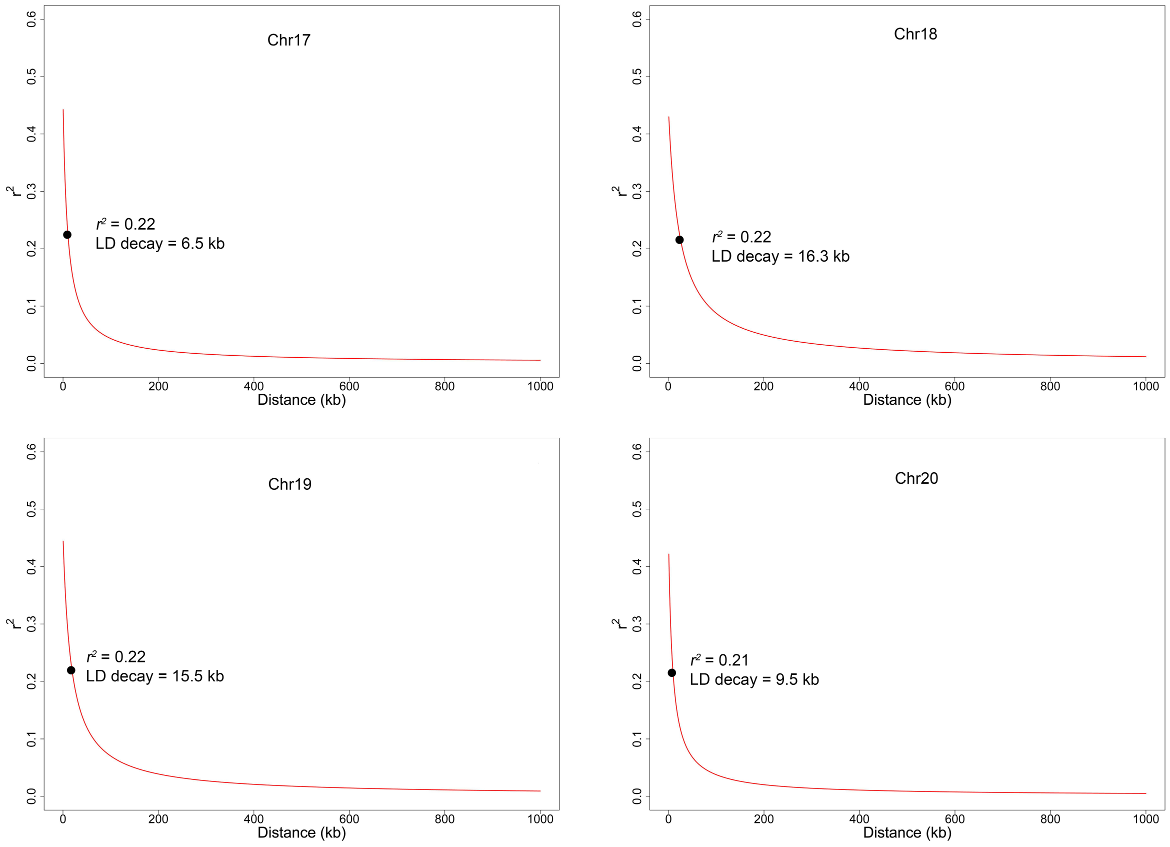
Chr17
r^2 = 0.22
LD decay = 6.5 kb
Chr18
r^2 = 0.22
LD decay = 16.3 kb
Chr19
r^2 = 0.22
LD decay = 15.5 kb
Chr20
r^2 = 0.21
LD decay = 9.5 kb
Distance (kb)
r^2
